# Supplementary material for: Anti-infective therapy using species-specific activators of Staphylococcus aureus ClpP
Source: Nat Commun. 2022 Nov 14;13:6909. doi: 10.1038/s41467-022-34753-0 (PMC9663597; doi:10.1038/s41467-022-34753-0)
Supplement: Supplementary file 1 — Supplementary Information [file 41467_2022_34753_MOESM1_ESM.pdf]

# Supplementary Information

# Anti-infective therapy using species-specific activators of *Staphylococcus aureus* ClpP

Bingyan Wei[1,2,3,#], Tao Zhang[2,#], Pengyu Wang[1,2,3,#], Yihui Pan[1,2,3], Jiahui Li[2,3], Weizhong Chen[4], Min Zhang[5], Quanjiang Ji[4], Wenjuan Wu[5], Lefu Lan[1,2,3], Jianhua Gan[6], Cai-Guang Yang[1,2,3,*]

Affiliations:

1. School of Pharmaceutical Science and Technology, Hangzhou Institute for Advanced Study, University of Chinese Academy of Sciences, Hangzhou 310024, China
2. State Key Laboratory of Drug Research, Centre for Chemical Biology, Shanghai Institute of Materia Medica, Chinese Academy of Sciences, Shanghai 201203, China.
3. University of Chinese Academy of Sciences, Beijing 100049, China.
4. School of Physical Science and Technology, ShanghaiTech University, Shanghai 201210, China.
5. Department of Laboratory Medicine, Shanghai East Hospital, Tongji University School of Medicine, Shanghai 200123, China.
6. School of Life Sciences, Fudan University, Shanghai 200433, China

#. These authors contributed equally to this work.

*. Correspondence, yangcg@simm.ac.cn (C.-G.Y.)

**Supplementary Table 1. Data collection and refinement statistics[a].**

| | ZG180/ *Sa*ClpP (7WID) | ZG180/ *Hs*ClpP (7WH5) | (*R*)-ZG197/ *Sa*ClpP (7XBZ) | (*S*)-ZG197/ *Sa*ClpP (7WGS) |
|---|---|---|---|---|
| **Data collection** | | | | |
| Space group | P 1 21 1 | P 1 21 1 | P 1 21 1 | P 1 21 1 |
| Cell dimensions | | | | |
| *a*, *b*, *c* (Å) | 94.2, 126.4, 145.5 | 111.9, 97.0, 131.6 | 93.9, 125.7, 145.2 | 94.0, 125.3, 145.9 |
| $\alpha$, $\beta$, $\gamma$ (°) | 90.0, 94.1, 90.0 | 90.0, 93.7, 90.0 | 90.0, 93.7, 90.0 | 90.0, 93.8, 90.0 |
| Resolution (Å) [b] | 30.0-1.90 (1.97-1.90) | 54.38-2.13 (2.19-2.13) | 30.0-2.15 (2.23-2.15) | 145.60-2.11 (2.22-2.11) |
| No. of observations | 1787498 | 1036870 | 618283 | 1006186 |
| No. unique | 266629 | 156976 | 171564 | 190050 |
| $R_{merge}$ [c] | 0.110 (0.799) | 0.112 (1.131) | 0.115 (0.285) | 0.095 (0.736) |
| $I/\sigma(I)$ | 16.0 (2.0) | 6.0 (1.2) | 12.9 (2.1) | 11.1 (2.6) |
| Completeness (%) | 98.9 (90.7) | 100.0 (100.0) | 94.4 (91.6) | 97.6 (99.1) |
| Redundancy | 6.7 (5.2) | 6.6 (6.5) | 3.6 (2.9) | 5.3 (4.0) |
| **Data refinement** | | | | |
| Resolution (Å) | 30.0-1.90 | 54.33-2.13 | 30.0-2.15 | 145.60-2.11 |
| No. reflections | 243547 | 148823 | 162704 | 180268 |
| $R_{work}/R_{free}$ | 0.197/0.206 | 0.230/0.249 | 0.196/0.221 | 0.187/0.203 |
| No. atoms | | | | |
| protein | 19875 | 18312 | 19368 | 19531 |
| ligand | 518 | 518 | 456 | 532 |
| water | 1557 | 254 | 491 | 749 |
| Average B value (Å$^2$) | | | | |
| protein | 20.71 | 52.69 | 37.97 | 41.05 |
| ligand | 23.46 | 75.37 | 67.12 | 59.67 |
| water | 29.60 | 44.66 | 34.73 | 43.14 |
| RMSD[d] in | | | | |
| Bond lengths (Å) | 0.010 | 0.012 | 0.011 | 0.010 |
| Bond angles (°) | 1.637 | 1.644 | 1.474 | 1.447 |
| Ramachandran | | | | |
| Preferred (%) | 98.6 | 97.4 | 97.8 | 97.7 |
| Allowed (%) | 1.4 | 2.6 | 2.2 | 2.3 |

[a] The structure was resolved using a single crystal.

[b] Highest resolution shell is shown in parenthesis.

[c] $R_{merge} = \Sigma|(I - \langle I \rangle)|/\Sigma(I)$, where I is the observed intensity.

[d] Root mean squared deviation.

**Supplementary Table 2. Information about the bacteria strains used in this work.**

| Strain | Description | Source |
|---|---|---|
| 8325-4 | Wild-type, *S. aureus* NCTC 8325-4 | [1] |
| 8325-4/Δ*clpP* | *clpP* deficient strain | [2] |
| 8325-4/Δ*clpP*/pYJ335::*clpP* | Δ*clpP* carrying pYJ335::*clpP* | This work |
| 8325-4/Δ*clpP*/pYJ335::*clpPI91W* | Δ*clpP* carrying pYJ335:: *clpPI91W* | This work |
| Newman | Wild-type, *S. aureus* ATCC 25904 | [3] |
| Newman/Δ*clpP* | *clpP* deficient strain | [4] |
| RN4220 | Derivative of 8325-4 that accepts plasmids | [5] |
| USA300 | A representative CA-MRSA isolate, which was first isolated in Los Angeles, Calif. | [6] |
| NRS1 | A HA-MRSA/VISA strain isolated in Japan | [7] |
| NRS70 | A HA-MRSA strain isolated in Japan | [7] |
| NRS100 | A HA-MRSA strain isolated in United States | [8] |
| NRS108 | A HA-MRSA strain isolated in France | [9] |
| NRS271 | A HA-MRSA strain isolated in United Kingdom | [10] |
| XJ009 | A HA-MRSA strain isolated in China | This work |
| XJ036 | A HA-MRSA strain isolated in China | This work |
| XJ049 | A HA-MRSA strain isolated in China | This work |
| XJ051 | A HA-MRSA strain isolated in China | This work |
| XJ052 | A HA-MRSA strain isolated in China | This work |
| DH5α | *E. coli* plasmid cloning host | Lab stock |
| BL21 (DE3) Gold | *E. coli* plasmid expression host | Lab stock |
| Rosetta (DE3) | *E. coli* plasmid expression host | Lab stock |

**Supplementary Table 3. Identification of spontaneous resistance mutants.**

| Clone | (*R*)-ZG197 | MIC (μg/mL) | (*S*)-ZG197 | MIC (μg/mL) |
|---|---|---|---|---|
| RC1 | 133A-V | >128 | 135E-K | >64 |
| RC2 | 55D-V | >128 | 53A-V | 32 |
| RC3 | GAP | >128 | 165I-T | >128 |
| RC4 | 101S-T | >128 | 140A-V | >128 |
| RC5 | 74G-V | >128 | 123H-N | >128 |
| RC6 | 124Q-R | >128 | 111R-C | >128 |
| RC7 | 116P-L | 32 | 72T-I | >128 |
| RC8 | 124Q-END | >128 | 123H-N | >128 |
| RC9 | 133A-V | >128 | 142H-Y | >128 |
| RC10 | 129A-T | 8 | 147R-P | >128 |
| RC11 | 157R-P | >128 | 72T-I | >128 |
| RC12 | 142H-Y | >128 | 86P-H | >128 |

## Supplementary Figures

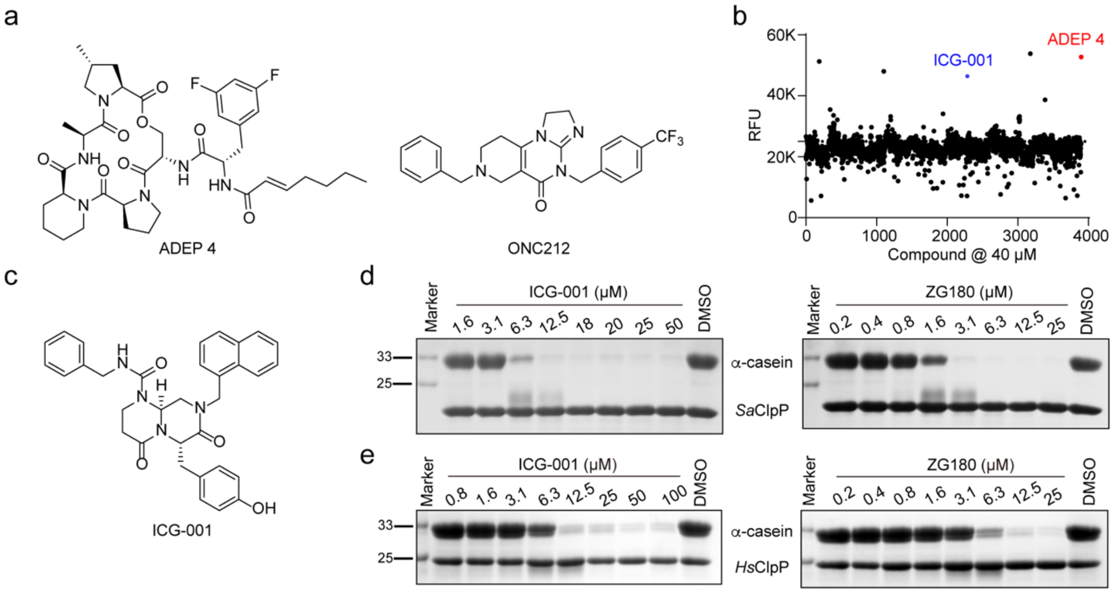


**Supplementary Fig. 1 High-throughput screening (HTS) and evaluation of effects of ClpP activators on ClpP proteolysis activity. a** Chemical structure of the global ClpP activators. **b** The HTS for *Sa*ClpP activators using the fluorogenic FITC-casein as a substrate of *Sa*ClpP. ADEP 4 was assayed as a positive control. The relative fluorescence units (RFU) were recorded and presented as mean values from duplicates. Five hit compounds were obtained and the hit rate is 0.13%. **c** The hit compound ICG-001 as a ClpP activator in this study. **d**, **e** Representative PAGE images for the detection of α-casein hydrolysis by *Sa*ClpP (**d**) and *Hs*ClpP (**e**) in the presence of ICG-001 and ZG180 at indicated concentrations (n = 3 biologically independent experiments).

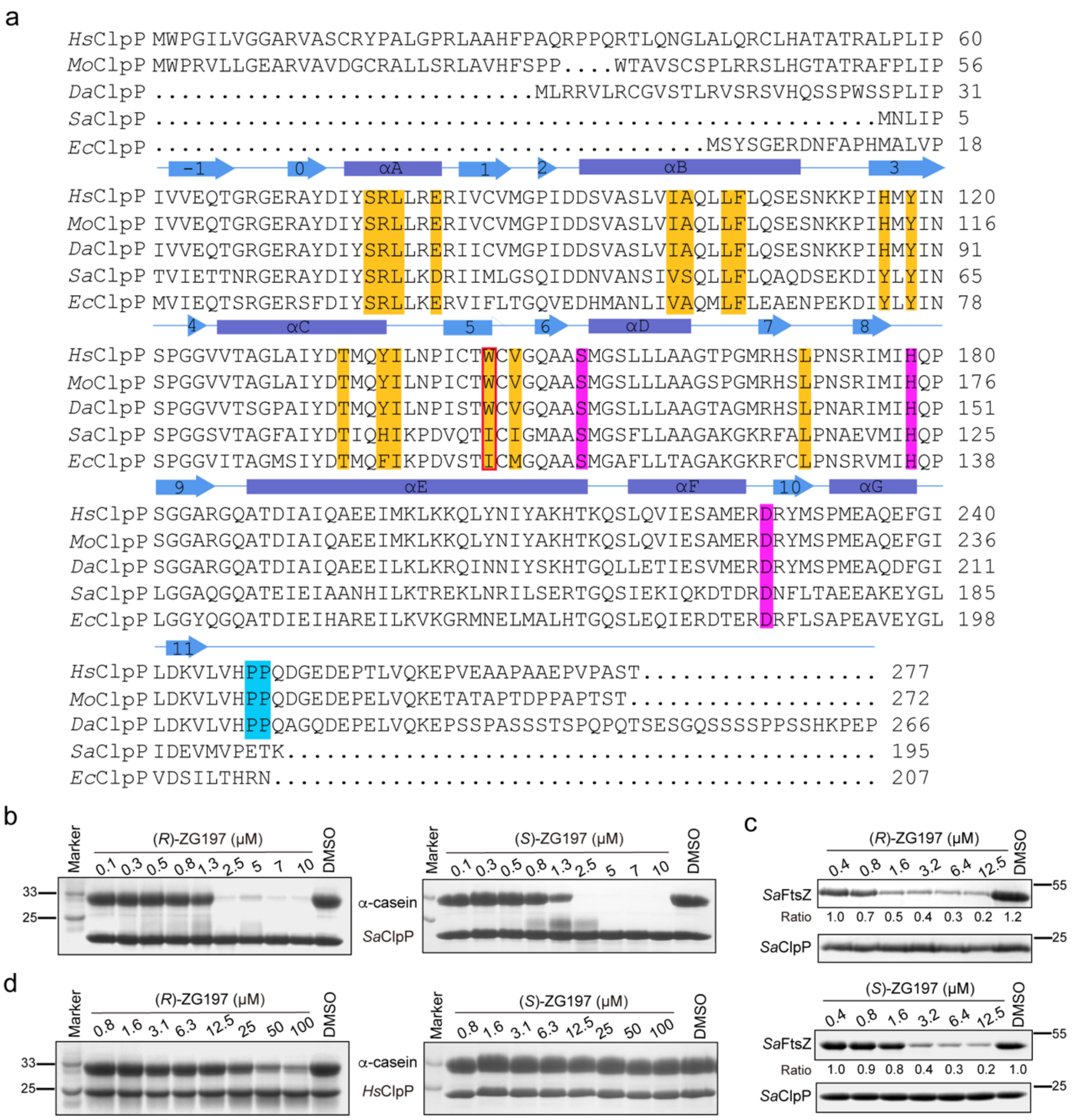


**Supplementary Fig. 2 Development of *Sa*ClpP-specific activators. a** Amino acid sequence alignment of *Hs*ClpP, *Mus Musculus* ClpP (*Mo*ClpP), *Danio Rerio* ClpP (*Da*ClpP), *Sa*ClpP, and *Escherichia coli* ClpP (*Ec*ClpP) performed in ClustalW2 and ESPript. Amino acid sequence can be found at National Center for Biotechnology Information (NCBI) with the accession number of NP_006003 [https://www.ncbi.nlm.nih.gov/protein/NP_006003.1] for *Hs*ClpP, CAA06443 [https://www.ncbi.nlm.nih.gov/protein/CAA06443.1] for *Mo*ClpP, NP_001018520 [https://www.ncbi.nlm.nih.gov/protein/NP_001018520.1] for *Da*ClpP, KFL07692 [https://www.ncbi.nlm.nih.gov/protein/KFL07692.1] for *Sa*ClpP, and CAD6014684 [https://www.ncbi.nlm.nih.gov/protein/CAD6014684.1] for *Ec*ClpP. The identical or highly simialr residues in the hydrophobic binding pockets are highlighted in orange,

the catalytic triad are shown in magenta, and the C-terminus proline residues are highlighted in cyan. The W146 in *Hs*ClpP and I91 in *Sa*ClpP are highlighted by a box in red. **b**, **d** Representative PAGE images for showing α-casein hydrolysis by *Sa*ClpP (**b**) and *Hs*ClpP (**d**) in the presence of (*R*)- and (*S*)-ZG197, respectively. **c** Representative PAGE images for showing the activation effect of (*R*)- and (*S*)-ZG197 on the recombinant *Sa*ClpP protein for the recombinant *Sa*FtsZ degradation.

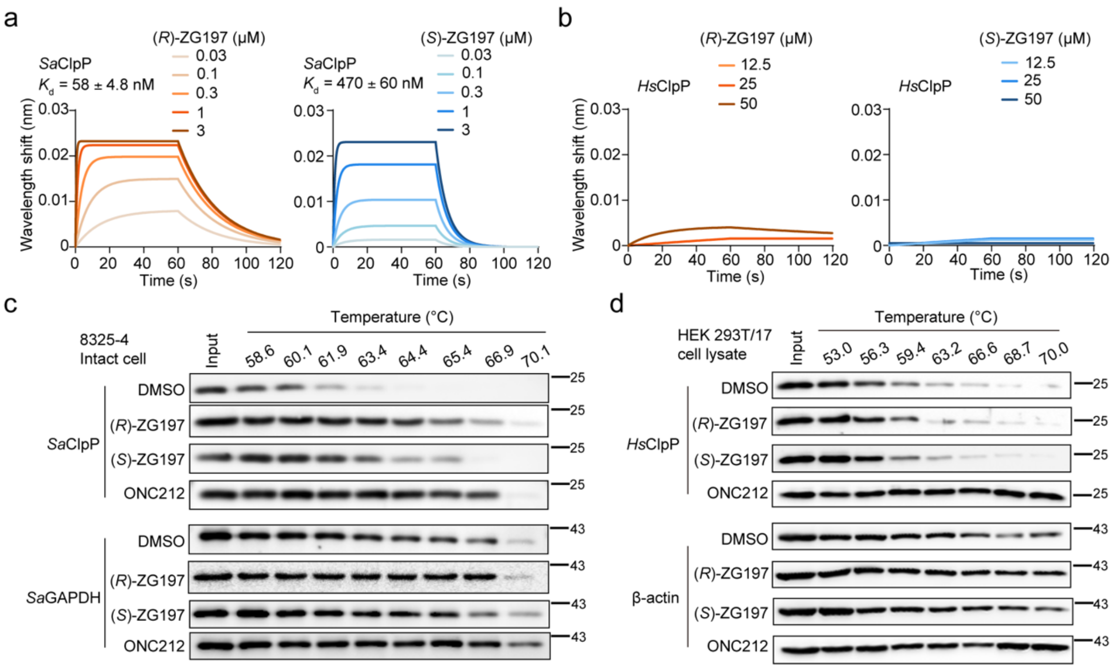


**Supplementary Fig. 3 Interactions between small-molecule activators and the ClpP proteases. a**, **b** Determination of the affinity of (*R*)- and (*S*)-ZG197 binding to *Sa*ClpP (**a**) and *Hs*ClpP (**b**) in the BLI assay. **c**, **d** Representative Western blot images showing the effect of 10 μM (*R*)-ZG197, (*S*)-ZG197, and ONC212 on the thermal stabilization of *Sa*ClpP in intact *S. aureus* 8325-4 strain (**c**) and *Hs*ClpP in the cell lysates of HEK 293T/17 cells (**d**), respectively. *Sa*GAPDH or β-actin was assayed as a loading control. The experiments were performed in three biological replicates.

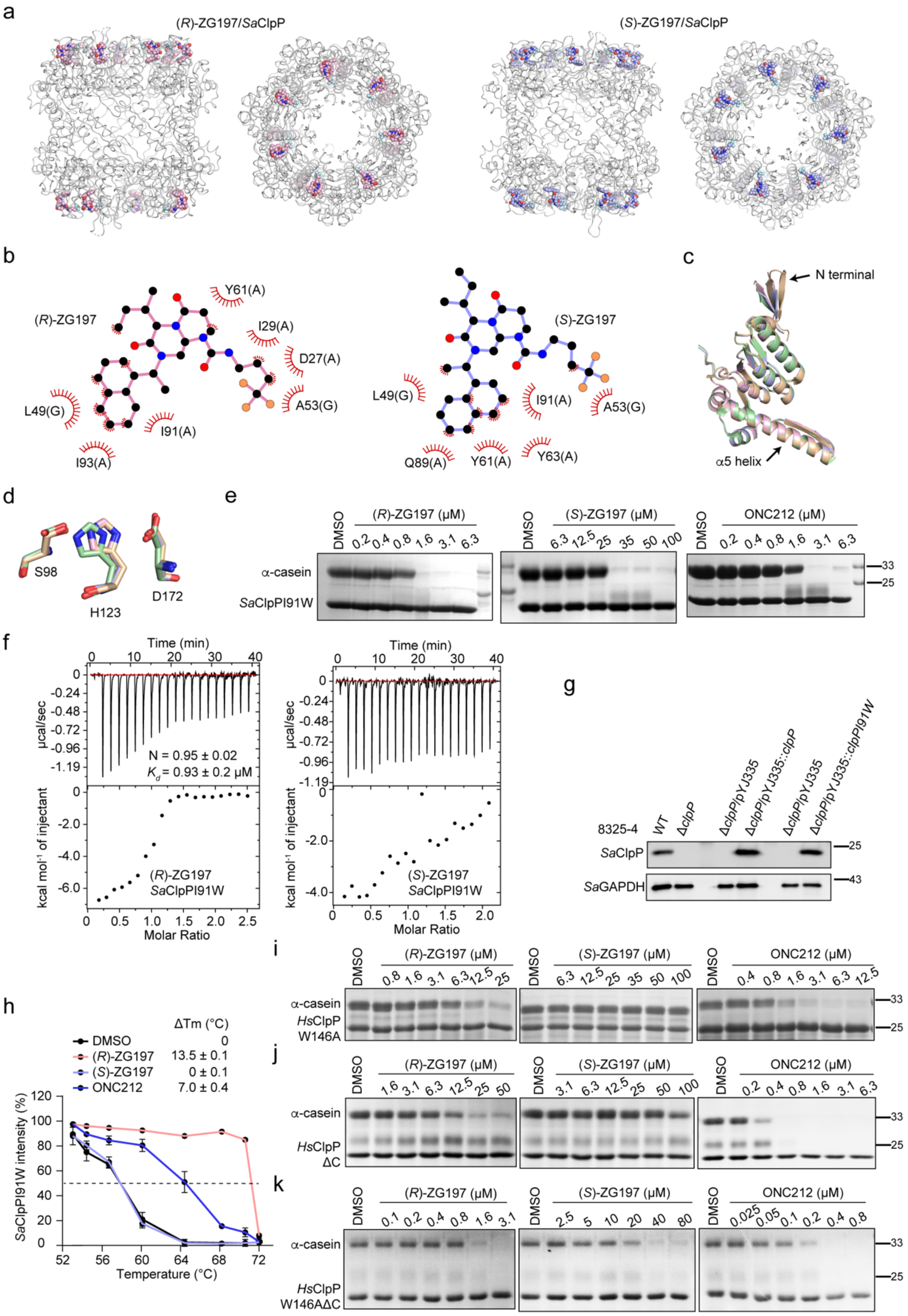


**Supplementary Fig. 4 Effect of small-molecule activators on ClpP variants. a** The resolved X-ray crystal structure of (*R*)-ZG197/*Sa*ClpP and (*S*)-ZG197/*Sa*ClpP from the side and top views. *Sa*ClpP is shown in gray cartoon tube and (*R*)- and (*S*)-ZG197 are shown in light pink and light blue spheres, respectively. **b** Representation of the main

hydrophobic interactions between *Sa*ClpP and (*R*)-ZG197 (left, light pink) or (*S*)-ZG197 (right, light blue) using Ligplot+ software. The atoms of carbon, oxygen, nitrogen and fluorine in (*R*)- and (*S*)-ZG197 are colored in black, red, blue and orange, respectively. The poked arcs represent residues making hydrophobic contacts with the compounds. **c** Superimposition of the monomers in the extended *Sa*ClpP (wheat, 3STA [http://doi.org/10.2210/pdb3STA/pdb] in PDB), ADEP 4/*Sa*ClpP (pale green, 6TTZ [http://doi.org/10.2210/pdb6TTZ/pdb] in PDB), (*R*)-ZG197/*Sa*ClpP (light pink, 7XBZ [http://doi.org/10.2210/pdb7XBZ/pdb] in PDB), and (*S*)-ZG197/*Sa*ClpP (light blue, 7WGS [http://doi.org/10.2210/pdb7WGS/pdb] in PDB). This yields low C$\alpha$ root mean square deviation (RMSD) values around 0.2 Å for the monomers. **d** Alignment of the catalytic triad in structural complexes showed in (**c**). **e** Representative PAGE images showing α-casein hydrolysis by the *Sa*ClpPI91W mutant in the presence of ClpP activators. **f** Determination of (*R*)- and (*S*)-ZG197 binding to the *Sa*ClpPI91W mutant protein in the ITC titration. **g** Representative PAGE images for showing cellular *Sa*ClpP expression in *S. aureus* 8325-4 strains with different genetic backgrounds of *clpP*, Δ*clpP*, and the complemented *clpP* or *clpI91W* mutant in Western blot analysis. *Sa*GAPDH was assayed as a loading control. Rescue of *Sa*ClpP or *Sa*ClpPI91W to the strain of *S. aureus* 8325-4/Δ*clpP* was achieved using a tetracycline-inducible vector pYJ335 in the presence of 1 ng/mL ATC. **h** Effect of activators on the thermal stability of the cellular *Sa*ClpPI91W protein (n = 3 biologically independent experiments). The Δ*clpP* mutant with the rescued *Sa*ClpPI91W was cultured in the presence of a 10 μM compound for 2 h before running CETSA. The proteins in unheated samples were used as inputs and considered as 100%. Data are shown as mean $\pm$ SD. **i, j, k** Representative PAGE images for showing α-casein hydrolysis by the *Hs*ClpPW146A mutant (**i**), the *Hs*ClpPΔC truncation (**j**) and the *Hs*ClpPW146AΔC truncation (**k**) in the presence of activators.

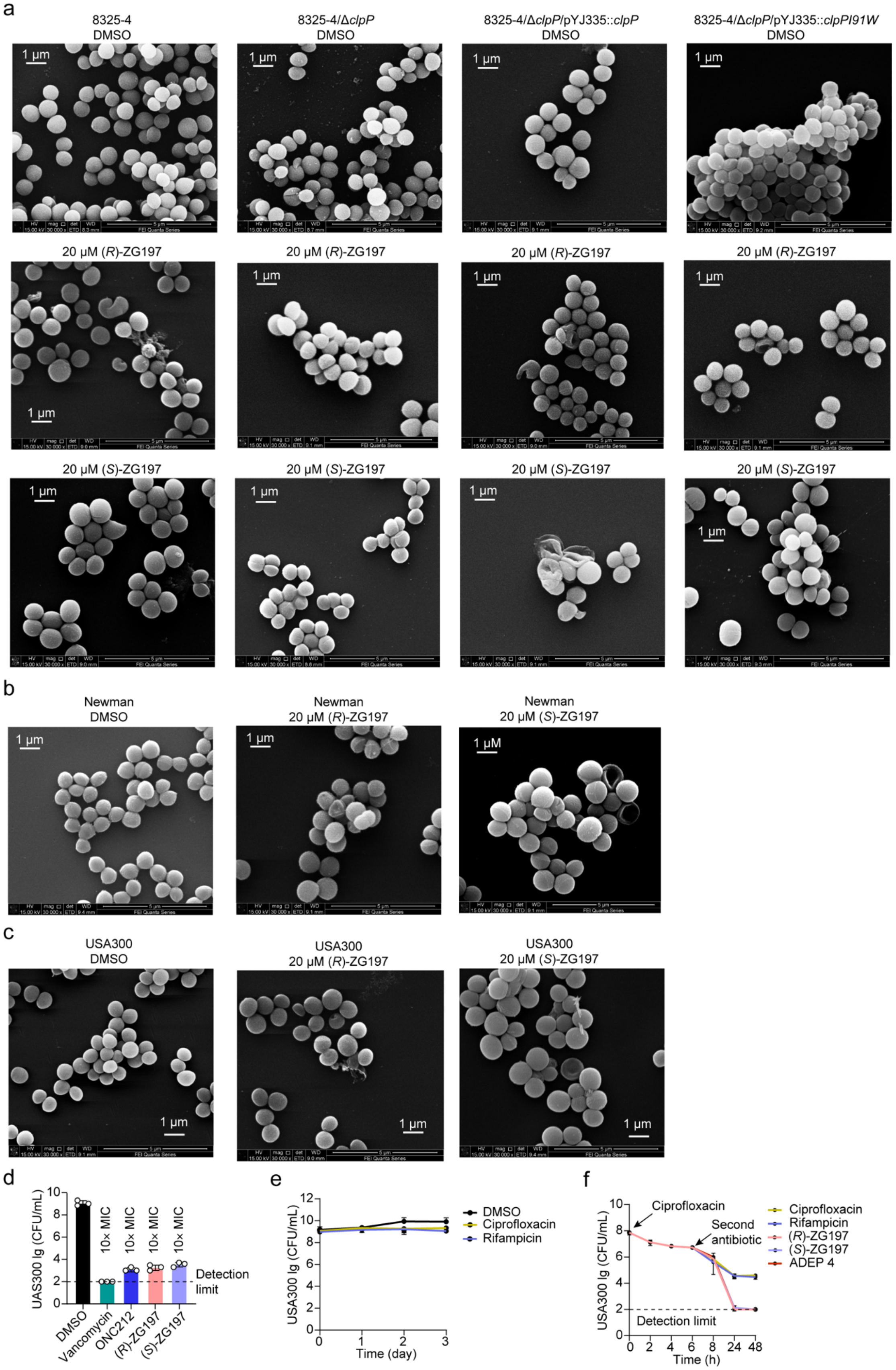


**Supplementary Fig. 5 Effect of our activators on cell morphology of *S. aureus* strains.**

**a** Full SEM images showing the cell morphology of *S. aureus* 8325-4 strains bearing

different genetic backgrounds of *clpP*, Δ*clpP*, and the complemented *clpP* or I91W mutant in the presence of (*R*)- and (*S*)-ZG197. **b**, **c** Full SEM images of the cell morphology of *S. aureus* Newman (**b**) and *S. aureus* USA300 (**c**) when treated with (*R*)- and (*S*)-ZG197. **d** Viability of *S. aureus* USA300 in exponential-phase when treated with vancomycin (20 μg/mL, 10 × MIC), (*R*)-ZG197 (5 μg/mL, 10 × MIC), (*S*)-ZG197 (80 μg/mL, 10 × MIC), or ONC212 (5 μg/mL, 10 × MIC) for 6 h. The limit of detection is indicated. CFU counts of bacteria were measured. **e** Effect of the conventional antibiotics on the growth of *S. aureus* USA300 in stationary phase. Rifampicin and ciprofloxacin were assayed at 0.4 μg/mL and 5 μg/mL, respectively. **f** Effects of *Sa*ClpP activators on the survival of *S. aureus* persisters after ciprofloxacin treatment. Data (**d**-**f**) are obtained from three biologically independent experiments and presented as mean ± SD (error bars).

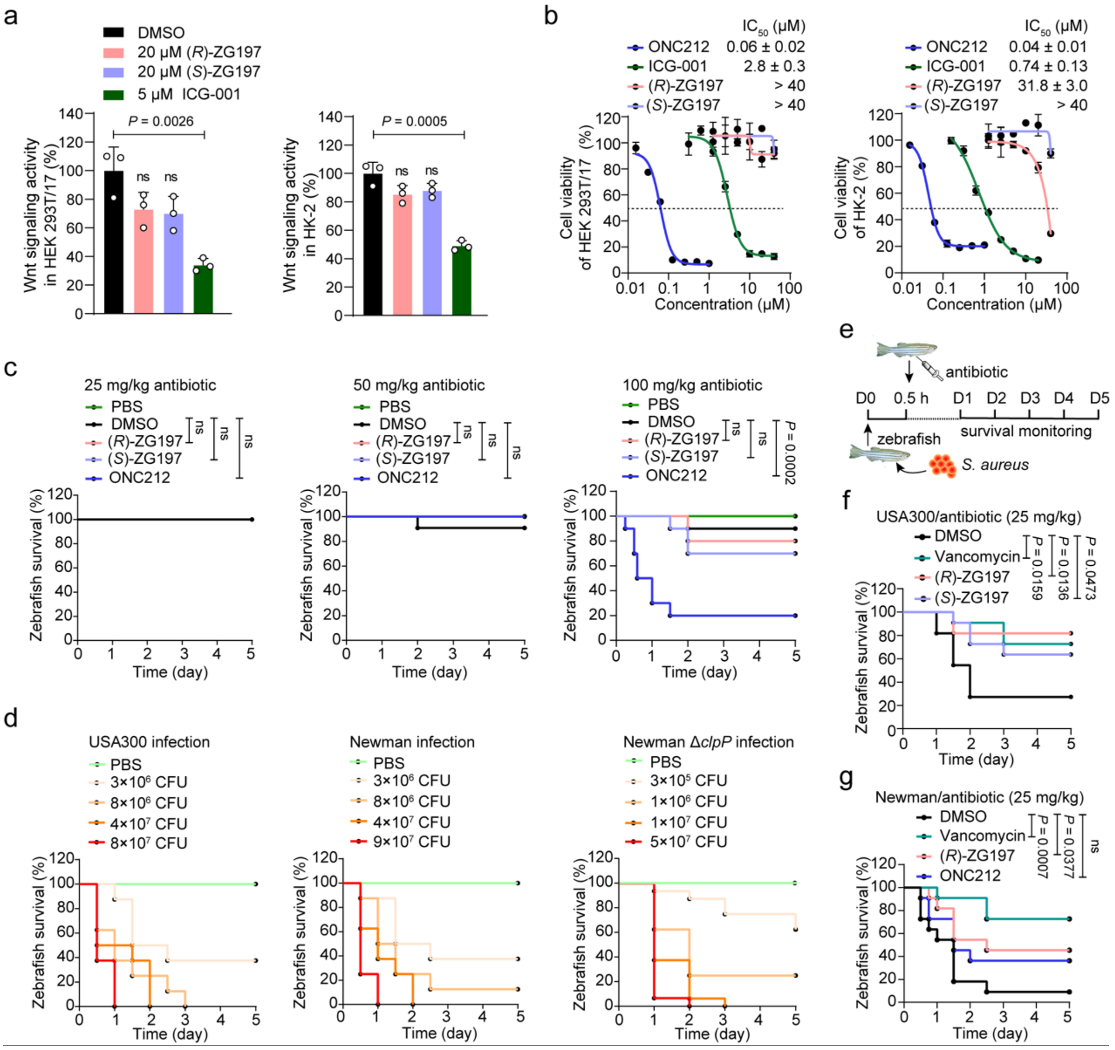


**Supplementary Fig. 6 Our activators exert bactericidal efficacy *in vivo* with minimal cytotoxic effects on mammalian cells. a** Effects of *Sa*ClpP activators on Wnt signaling activity in HEK 293T/17 (left) and HK-2 (right) cell lines using Wnt/β-catenin Luciferase reporter assay. **b** Inhibitory effect of *Sa*ClpP activators on cell viability in HEK 293T/17 (left) and HK-2 (right) cell lines detected in MTT assay. **c** Assessment of the cytotoxic effect of compounds on zebrafish (n = 11 animals). Compounds were administered at dosages of 25, 50 or 100 mg/kg, and the survival of zebrafish was recorded. **d** Effects of colony-forming units (CFUs) of the *S. aureus* strains on the mortality of infected zebrafish (n = 8 animals). **e** Scheme illustration of evaluation on the *in vivo* antibacterial effects of our activators on zebrafish infected with *S. aureus*. **f** The therapeutic effect of (*R*)- and (*S*)-ZG197 on *S. aureus* USA300 infection. Zebrafish (n = 11 animals) were infected with $8 \times 10^6$ CFU of *S. aureus* USA300, and the compound was administered

at a single dosage of 25 mg/kg. Vancomycin was tested as a control, and ONC212 was similarly administered. **g** Therapeutic effect of (*R*)-ZG197 and ONC212 against *S. aureus* Newman infection. Zebrafish (n = 11 animals) were infected with $4 \times 10^7$ CFU of *S. aureus* Newman, and the compound was administered at a single dosage of 25 mg/kg. Vancomycin was tested as a control. Statistical differences were analyzed using two-tailed unpaired Student's t-tests (**a**) and Mantel-Cox tests (**c**, **f** and **g**). Exact *P* values are provided. ns, no significance. Data (**a** and **b**) are obtained from three biologically independent experiment and are presented as mean ± SD (error bars).

## Supplementary methods

### General Information.

All solvents and reagents were purchased from commercial vendors and used without purification. Reactions were monitored using Agilent 1200 (HPLC) and 6110 (MSD) systems with Xbridge C18, 3.5 μm, 4.6 × 50 mm column. HPLC gradient method utilized a 5% to 95% acetonitrile in $H_2O$ with 0.01% trifluoroacetic acid over 5 min with a 1.0 mL/min flow rate. Final products were purified by reverse-phase preparatory using a Lisui EZ Plus 100 D with a PDA detector and Kromat flash C18 20 - 40 μm 120 g column. Purification methods used a 30 min gradient from 20% to 80% acetonitrile in $H_2O$. $^1H$ NMR spectra were recorded on Bruker-400 (400 MHz) and Bruker-500 (500 MHz) spectrometers using $CDCl_3$ as solvent. $^{13}C$ NMR spectra were detected on Bruker-500 (125 MHz) spectrometer using $CDCl_3$ as solvent. The purity of the final compound was assessed at a wavelength of 254 nm by HPLC analysis. The purities of all target compounds are >95% in HPLC. All final compounds were additionally freeze-dried before use in biochemical and cellular experiments. Compound names were generated using ChemBioDraw Ultra v14.0 systematic naming.

### Synthesis of 1,1,1-trifluoro-4-isocyanatobutane.

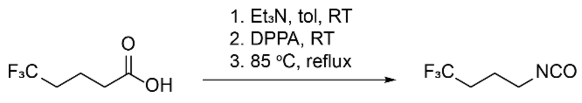

Triethylamine ($Et_3N$) (121 mg, 1.2 mmol, 166 µL, 1.2 eq) was added to a solution of 5,5,5-trifluoropentanoic acid (156 mg, 1.0 mmol, 1.0 eq) in toluene (5 mL) and stirred for 5 min at RT. Diphenyl phosphorazidate (DPPA) (303 mg, 1.1 mmol, 237 µL, 1.1 eq) was then added dropwise, stirred for an additional 1 h, and then refluxed for 2 h and finally cooled. The products obtained were not characterized because of their high instability and were directly used in the next reaction.

**Synthesis of ZG180.**

Compounds **3** and **8** were synthesized as in our previous study[11].

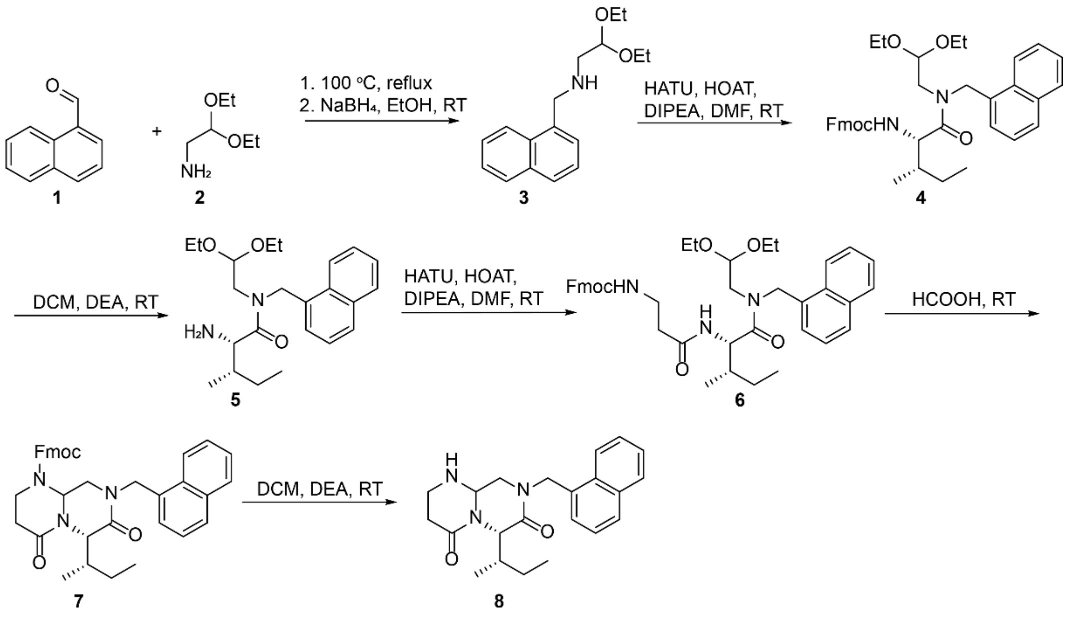


(6*S*,9a*S*)-6-((*S*)-sec-butyl)-8-(naphthalen-1-ylmethyl)-4,7-dioxo-*N*-(4,4,4-trifluorobutyl)hexahydro-2*H*-pyrazino[1,2-a]pyrimidine-1(6*H*)-carboxamide **ZG180**.

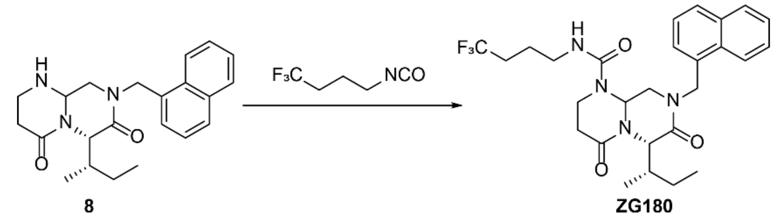


Compound **8** (73 mg, 0.2 mmol) was added to the cooled solution of 1,1,1-trifluoro-4-isocyanatobutane in toluene, then stirred for 6 h at RT. The mixture was concentrated *in vacuo* and the residue was purified by reversed-phase C18 (RP-C18) column chromatography to give **ZG180** as a white powder (86 mg). $[\alpha]^{D}_{20}$ = +80.5 (c = 0.4 g in

100 mL acetonitrile). $^1$H NMR (500 MHz, $CDCl_3$): δ 8.11 – 8.05 (m, 1H), 7.87 (m, 1H), 7.84 (d, 1H), 7.56 – 7.48 (m, 2H), 7.45 – 7.39 (t, *J* = 7.1 Hz, 1H), 7.34 (d, *J* = 6.9 Hz, 1H), 6.01 (dd, *J* = 9.5, 5.5 Hz, 1H), 5.21 (d, *J* = 6.25 Hz, 1H), 5.16 (d, *J* = 14.6 Hz, 1H), 5.02 (d, *J* = 14.6 Hz, 1H), 3.57 (dd, *J* = 14.2, 4.4 Hz, 1H), 3.34 – 3.11 (m, 5H), 2.51 (m, 1H), 2.33 (d, *J* = 16.9 Hz, 1H), 2.19 (m, 1H), 2.11 – 2.02 (m, 2H), 1.76 – 1.68 (m, 2H), 1.63 – 1.52 (m, 1H), 1.32 – 1.23 (m, 1H), 1.12 (d, *J* = 6.8 Hz, 3H), 0.93 (t, *J* = 7.3 Hz, 3H). $^{13}$C NMR (125 MHz, $CDCl_3$): δ 166.4, 165.8, 156.0, 134.0, 131.6, 131.3, 129.2, 128.8, 126.9, 126.3, 125.1, 123.8, 60.9, 59.6, 48.6, 48.2, 39.9, 38.5, 37.7, 31.5, 31.3, 26.3, 22.8, 22.7, 16.1, 12.0. LRMS (ESI+) *m/z*: $[M + H]^+$, 519.2; HRMS (m/z): $[M + H]^+$ calcd. for $C_{27}H_{33}F_3N_4O_3$ 519.2583, found 519.2574; HPLC purity at 280 nm, 98.4%. The solubility of **ZG180** was estimated to be 130 μM in PBS buffer in the presence of 1% DMSO.

**Synthesis of 2,2-diethoxyacetaldehyde 10.**

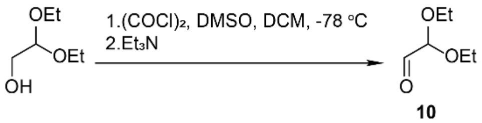


To a solution of oxalyl chloride (1.3 g, 10.5 mmol, 1.05 eq) in DCM (15 mL) was added dropwise DMSO (1.6 g, 21.0 mmol, 2.1 eq) in DCM (10 mL) at -78 °C over 30 min and the mixture was stirred at -78 °C. Then a solution of 2,2-diethoxyethan-1-ol (1.3 g, 10.0 mmol, 1.0 eq) in DCM (10 mL) was added dropwise and the solution was stirred at -78 °C. After 30 min, $Et_3N$ (5.1 g, 50.0 mmol, 5.0 eq) was added dropwise over 1 h, the mixture was stirred for 30 min at -78 °C and then warmed to RT over 30 min. The resulting mixture was washed with brine and concentrated *in vacuo* to afford the crude **10** (1.0 g) as a yellow oil. The product was used in the next step without further purification.

**Synthesis of (*R*)- and (*S*)-ZG197**

The synthesis of (*R*)-2,2-diethoxy-N-(1-(naphthalen-1-yl)ethyl)ethan-1-amine **11**.

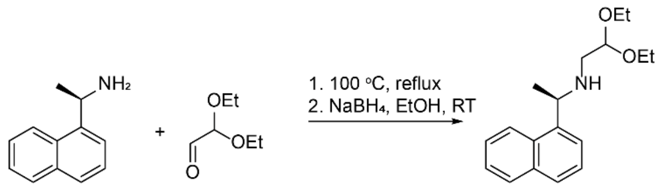

9 10 11

A mixture of (*R*)-1-(naphthalen-1-yl)ethan-1-amine **9** (1.3 g, 7.6 mmol, 1.0 eq) and 2,2-diethoxyacetaldehyde **10** was heated at 100 °C for 2 h, then cooled to RT and diluted with EtOH (25 mL). Sodium borohydride (578 mg, 15.2 mmol, 2.0 eq) was added in small portions and the mixture was stirred at RT for 24 h. The solvent was removed under reduced pressure, the residue was diluted with DCM (20 mL) and sequentially washed with water and brine. The organic phase was dried over $Na_2SO_4$, concentrated under reduced pressure and purified by gel column chromatography, eluting with petroleum ether (PE)/ethyl acetate (EA) (10:1) to afford the product **11** as a yellow oil (1.3 g). $^1$H NMR (500 MHz, $CDCl_3$): δ 8.22 (d, *J* = 8.4 Hz, 1H), 7.93 – 7.83 (m, 1H), 7.76 (d, *J* = 8.2 Hz, 1H), 7.70 (d, *J* = 7.1 Hz, 1H), 7.55 – 7.40 (m, 3H), 4.66 (dt, *J* = 11.0, 6.0 Hz, 2H), 3.75 – 3.61 (m, 2H), 3.60 – 3.45 (m, 2H), 2.74 (qd, *J* = 12.0, 5.5 Hz, 2H), 1.53 (d, *J* = 6.6 Hz, 3H), 1.23 (t, *J* = 7.1 Hz, 3H), 1.19 (t, *J* = 7.1 Hz, 3H). $^{13}$C NMR (125 MHz, $CDCl_3$): δ 140.6, 133.6, 130.9, 128.5, 126.8, 125.3, 124.9, 122.5, 122.4, 101.9, 62.0, 61.6, 53.1, 49.8, 23.2, 15.0, 15.0. LRMS (ESI+) *m/z*: $[M + H]^+$, 288.2.

(6*S*)-6-((*S*)-sec-butyl)-8-((*R*)-1-(naphthalen-1-yl)ethyl)hexahydro-4H-pyrazino[1,2-a]pyrimidine-4,7(6H)-dione **16 (*R*)-intermediate** was synthesized as in our previous study[11].

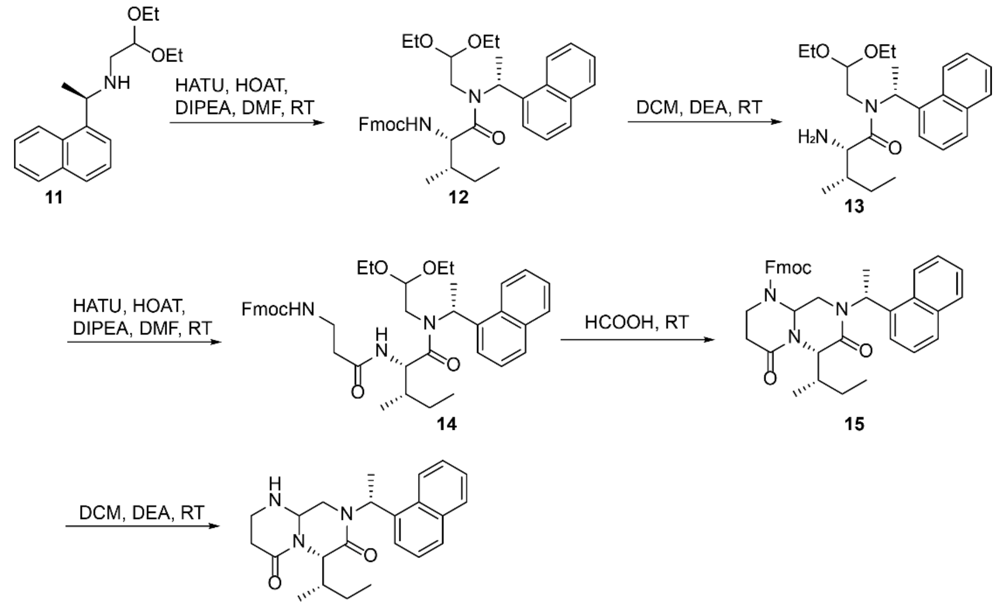


**16 (*R*)-Intermediate**

**16 (*R*)-intermediate** was obtained as a white powder. $^{1}$H NMR (400 MHz, $CDCl_3$) δ 8.14 – 8.04 (m, 1H), 7.96 – 7.81 (m, 2H), 7.51 (m, 4H), 6.72 (q, $J$ = 6.8 Hz, 1H), 5.21 (d, $J$ = 6.1 Hz, 1H), 4.37 (dd, $J$ = 9.3, 4.0 Hz, 1H), 3.20 – 3.03 (m, 2H), 3.02 – 2.90 (m, 1H), 2.70 (dd, $J$ = 12.1, 4.1 Hz, 1H), 2.42 (t, $J$ = 6.2 Hz, 2H), 2.07 – 1.96 (m, 1H), 1.67 (m, 4H), 1.55 (m, 1H), 1.22 – 1.15 (m, 1H), 1.11 (d, $J$ = 6.8 Hz, 3H), 0.93 (t, $J$ = 7.3 Hz, 3H). $^{13}$C NMR (125 MHz, $CDCl_3$) δ 167.1, 166.5, 134.7, 133.9, 131.7, 129.0, 128.7, 126.5, 126.2, 125.4, 124.9, 124.0, 64.6, 58.8, 47.6, 45.0, 39.1, 38.6, 33.5, 26.9, 16.2, 15.4, 12.0. LRMS (ESI+) *m/z*: $[M + H]^+$, 380.2.

The synthesis of (6*S*)-6-((*S*)-sec-butyl)-8-((*R*)-1-(naphthalen-1-yl)ethyl)-4,7-dioxo-N-(4,4,4-trifluorobutyl)hexahydro-2H-pyrazino[1,2-a]pyrimidine-1(6H)-carboxamide **(*R*)-ZG197**.

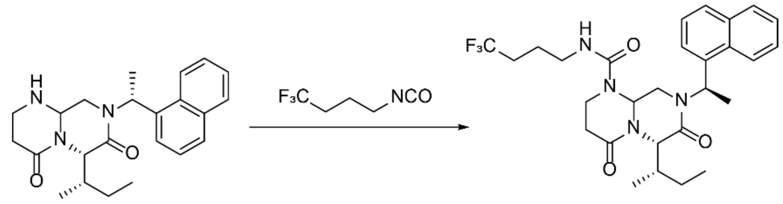


**16 (*R*)-Intermediate** **(*R*)-ZG197**

**16 (*R*)-intermediate** (76 mg, 0.2 mmol) was added to the prepared solution of 1,1,1-trifluoro-4-isocyanatobutane in toluene, then stirred for 6 h at RT. The mixture was concentrated *in vacuo* and the residue was purified by reversed-phase C18 (RP-C18)

column chromatography to give ***R*)-ZG197** as a white powder (87 mg). $[\alpha]_{20}^{D}$ = +52.1 (c = 0.2 g in 100 mL acetonitrile). $^{1}$H NMR (500 MHz, $CDCl_3$) δ 8.10 – 8.03 (m, 1H), 7.89 (m, 1H), 7.85 (d, *J* = 7.9 Hz, 1H), 7.57 – 7.42 (m, 4H), 6.70 (q, *J* = 6.9 Hz, 1H), 5.42 (dd, *J* = 10.7, 3.8 Hz, 1H), 5.23 (d, *J* = 5.8 Hz, 1H), 4.13 (t, *J* = 5.4 Hz, 1H), 3.76 (dd, *J* = 14.3, 5.0 Hz, 1H), 3.34 – 3.22 (m, 2H), 3.15 – 2.99 (m, 2H), 2.52 (m, 2H), 2.44 – 2.36 (m, 1H), 2.22 – 2.12 (m, 1H), 2.01 – 1.91 (m, 2H), 1.65 (d, *J* = 6.9 Hz, 3H), 1.56 (m, 2H), 1.25 – 1.16 (m, 1H), 1.11 (d, *J* = 6.9 Hz, 3H), 0.93 (t, *J* = 7.3 Hz, 3H). $^{13}$C NMR (125 MHz, $CDCl_3$) δ 165.4, 165.3, 155.3, 134.0, 133.5, 131.0, 128.8, 128.5, 126.2, 125.9, 124.9, 124.4, 123.3, 61.4, 59.3, 47.7, 43.1, 39.4, 38.5, 37.0, 31.2, 30.8, 30.5, 25.9, 22.2, 15.6, 15.0, 11.6. LC-MS $[M + H]^{+}$ 533.3; HRMS [M + H]+ (ESI-TOF) calcd for $C_{28}H_{35}F_3N_4O_3$ 533.2740, found 533.2734; HPLC purity at 280 nm, 98.2%. The solubility of **(*R*)-ZG197** was estimated to be 127 μM in PBS buffer in the presence of 1% DMSO.

**(*S*)-ZG197** was synthesized with (*S*)-1-(naphthalen-1-yl)ethan-1-amine as starting material. The subsequent synthesis methods are the same with **(*R*)-ZG197**.

(6*S*)-6-((*S*)-sec-butyl)-8-((*S*)-1-(naphthalen-1-yl)ethyl)hexahydro-4H-pyrazino[1,2-a]pyrimidine-4,7(6H)-dione **(*S*)-intermediate.**

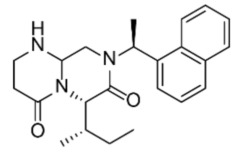

**(*S*)-intermediate**

**(*S*)-intermediate** was obtained as a white powder. $^{1}$H NMR (400 MHz, $CDCl_3$) δ 7.97 – 7.79 (m, 3H), 7.62 – 7.46 (m, 4H), 6.64 (q, *J* = 6.8 Hz, 1H), 5.14 (d, *J* = 6.7 Hz, 1H), 4.58 (s, 1H), 3.31 (dd, *J* = 13.1, 4.7 Hz, 1H), 2.77 (dd, *J* = 13.7, 6.6 Hz, 1H), 2.64 (dd, *J* = 13.5, 6.5 Hz, 1H), 2.44 (dd, *J* = 13.1, 6.9 Hz, 1H), 2.27 (dt, *J* = 17.2, 6.0 Hz, 1H), 2.11 – 1.96 (m, 2H), 1.73 – 1.55 (m, 5H), 1.37 – 1.25 (m, 2H), 1.11 (d, *J* = 6.8 Hz, 3H), 0.99 (t, *J* = 7.4 Hz, 3H), 0.78 (d, *J* = 8.4 Hz, 1H). $^{13}$C NMR (125 MHz, $CDCl_3$) δ 167.3, 167.2, 134.3, 133.8, 131.6, 129.2, 128.8, 127.4, 126.3, 125.3, 125.0, 123.4, 65.4, 58.7, 47.4, 44.7, 39.2, 38.3, 32.7, 27.1, 16.1, 16.0, 11.8. LC-MS $[M + H]^{+}$ 380.2.

(6*S*,9a*S*)-6-((*S*)-sec-butyl)-8-((*S*)-1-(naphthalen-1-yl)ethyl)-4,7-dioxo-*N*-(4,4,4-trifluorobutyl)hexahydro-2*H*-pyrazino[1,2-a]pyrimidine-1(6*H*)-carboxamide **(*S*)-**

**ZG197**.

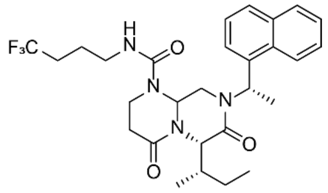

(*S*)-ZG197

**(*S*)-ZG197** was obtained as a white powder (81 mg). $[\alpha]_{20}^{D}$ = +99.1 (c = 0.2 g in 100 mL acetonitrile). $^{1}H$ NMR (500 MHz, $CDCl_3$) δ 7.91 (d, *J* = 8.4 Hz, 1H), 7.88 – 7.80 (m, 1H), 7.83 (d, *J* = 8.1 Hz, 1H), 7.58 – 7.49 (m, 3H), 7.48 – 7.43 (m, 1H), 6.63 (q, *J* = 6.8 Hz, 1H), 5.97 (dd, *J* = 10.2, 4.0 Hz, 1H), 5.19 (d, *J* = 5.5 Hz, 1H), 4.64 (t, *J* = 5.6 Hz, 1H), 3.35 – 3.21 (m, 3H), 3.13 (dd, *J* = 12.1, 4.0 Hz, 1H), 2.89 (ddd, *J* = 13.8, 11.5, 3.8 Hz, 1H), 2.44 – 2.31 (m, 2H), 2.29 – 2.19 (m, 1H), 2.18 – 2.05 (m, 3H), 1.77 (dt, *J* = 14.8, 7.3 Hz, 2H), 1.66 (t, *J* = 6.0 Hz, 3H), 1.40 – 1.29 (m, 1H), 1.11 (d, *J* = 6.9 Hz, 3H), 0.98 (t, *J* = 7.3 Hz, 3H). $^{13}C$ NMR (125 MHz, $CDCl_3$) δ 165.6, 165.3, 155.5, 133.6, 133.2, 131.3, 128.5, 128.2, 127.0, 125.8, 124.8, 124.4, 122.8, 61.5, 59.1, 47.4, 43.8, 39.4, 38.7, 37.4, 30.9, 30.9, 30.7, 30.5, 26.1, 22.3, 15.6, 15.4, 11.7. LC-MS $[M + H]^+$ 533.3; HRMS [M + H]+ (ESI-TOF) calcd for $C_{28}H_{35}F_3N_4O_3$ 533.2740, found 533.2735; HPLC purity at 280 nm, 99.3%. The solubility of **(*S*)-ZG197** was estimated to be 120 μM in PBS buffer in the presence of 1% DMSO.

**Determination of compound solubility**

The solubilities of compounds were determined by Agilent 1200 HPLC system furnished with an Xbridge C18 column (3.5 μm, 4.6 × 50 mm) and a photo-diode array ultraviolet detector at RT. The gradient method utilized a 5% to 95% acetonitrile in water in the presence of 0.01% trifluoroacetic acid over 5 min at a flow rate of 1.0 mL/min, and the ultraviolet absorption was recorded at 280 nm. The compounds in DMSO solution were then diluted to concentrations of 7.8, 15.6, 31.3, 62.5, 125, 250, 500, and 1000 μM. The standard curves of ZG180, (*R*)- and (*S*)-ZG197 were established with concentration of compound as X-axis and peak area as Y-axis, and the correlation coefficient ($r^2$) of the curves were 0.9993, 0.9994, and 0.9991, respectively. PBS (990

µL) and 10 µL 50 mM ZG180 or (*R*)- and (*S*)-ZG197 in DMSO were added in a colorless transparent glass bottle with magneton inside and stirred at 25 °C for 4 h. The turbid solution was then centrifuged at 10,000 rpm for 5 min and the supernatant was subjected to HPLC analysis. The concentrations of three compounds in the supernatants, namely the solubility of ZG180, (*R*)- and (*S*)-ZG197, were calculated based on the standard curves.

**NMR spectrum Data of ZG180, (*R*)- and (*S*)-ZG197**

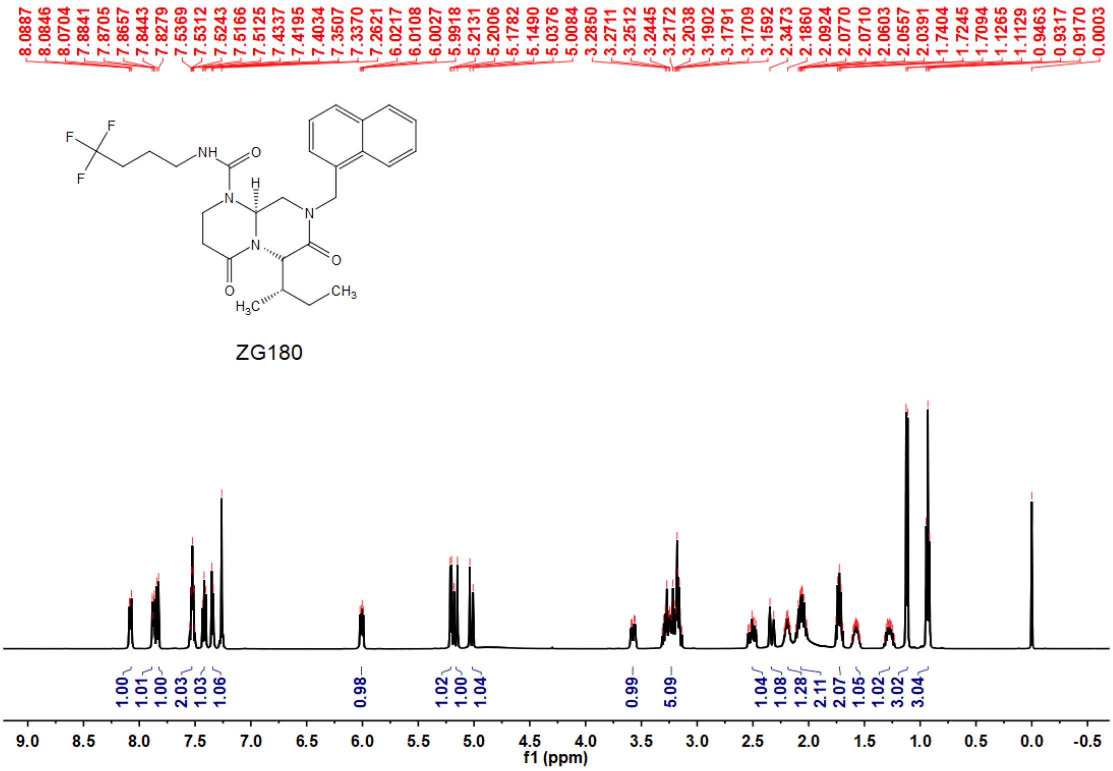


**Supplementary Fig. 7 $^{1}$H NMR-spectrum (500 MHz, $CDCl_3$) of ZG180**

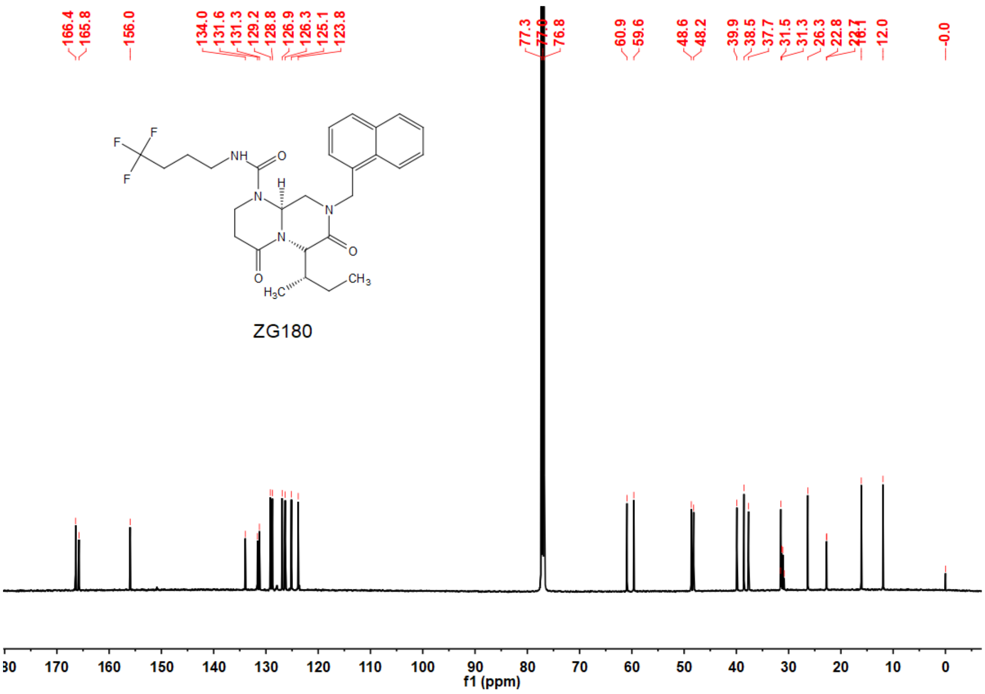


**Supplementary Fig. 8 $^{13}$C NMR-spectrum (125 MHz, $CDCl_3$) of ZG180**

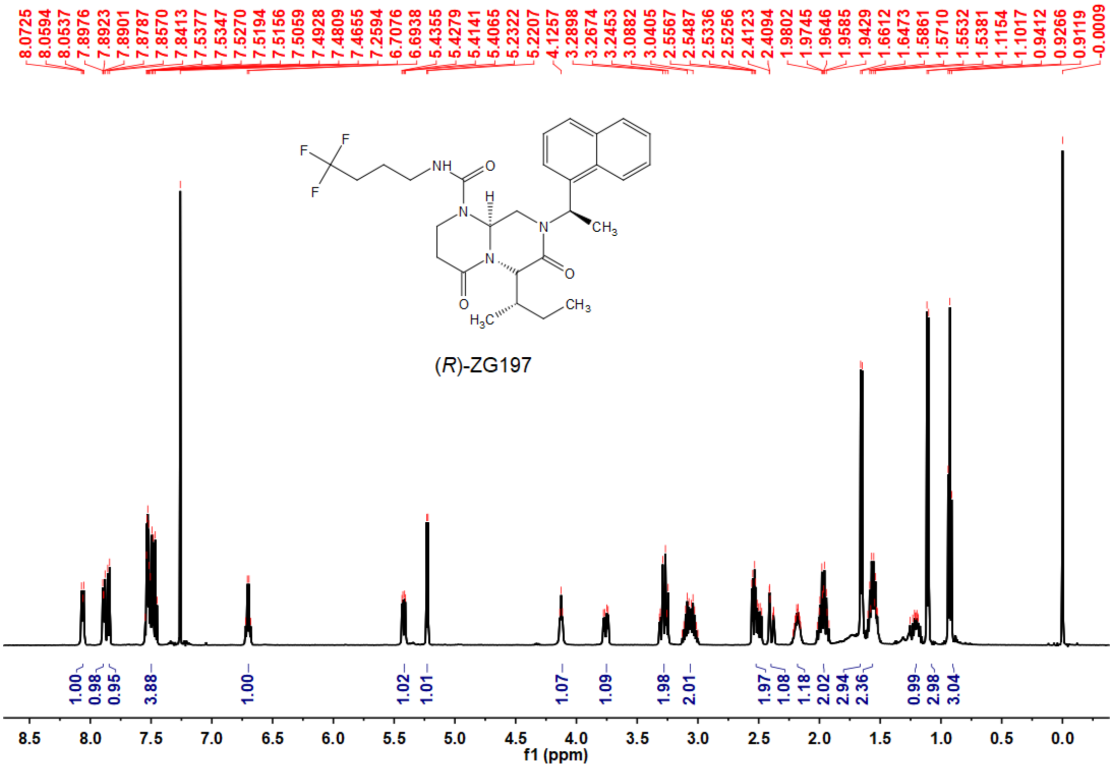


**Supplementary Fig. 9 $^{1}$H NMR-spectrum (500 MHz, $CDCl_3$) of (*R*)-ZG197**

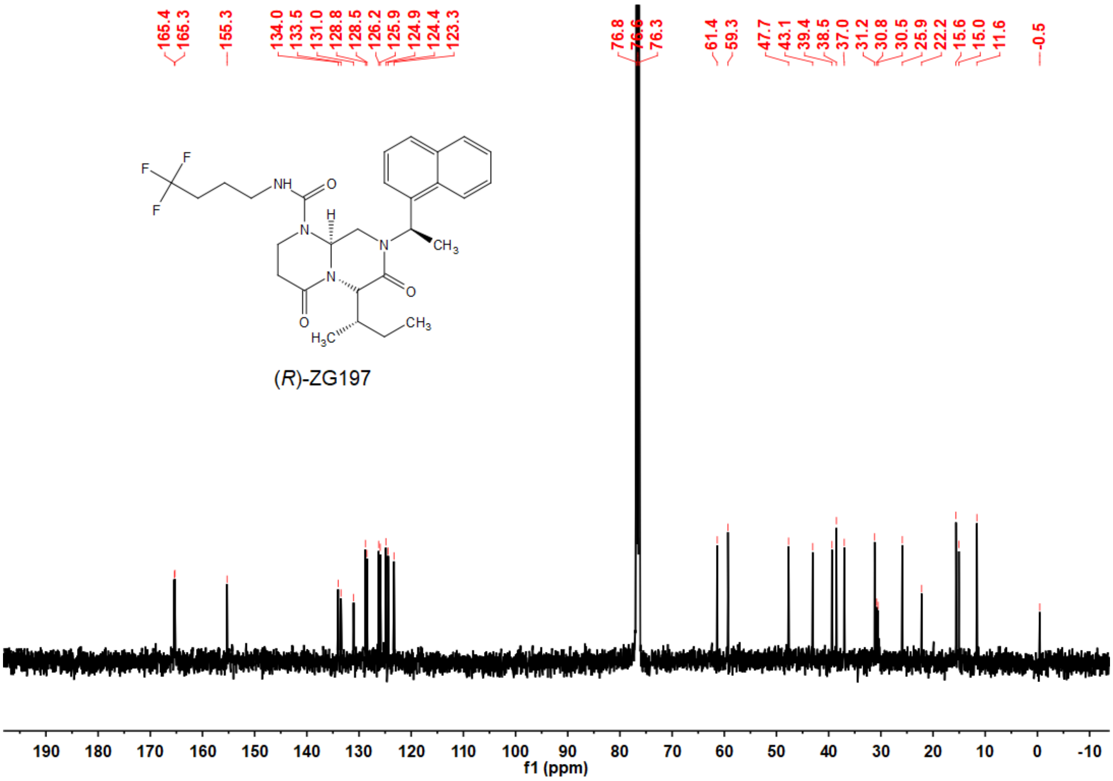


**Supplementary Fig. 10 $^{13}$C NMR-spectrum (125 MHz, $CDCl_3$) of (*R*)-ZG197**

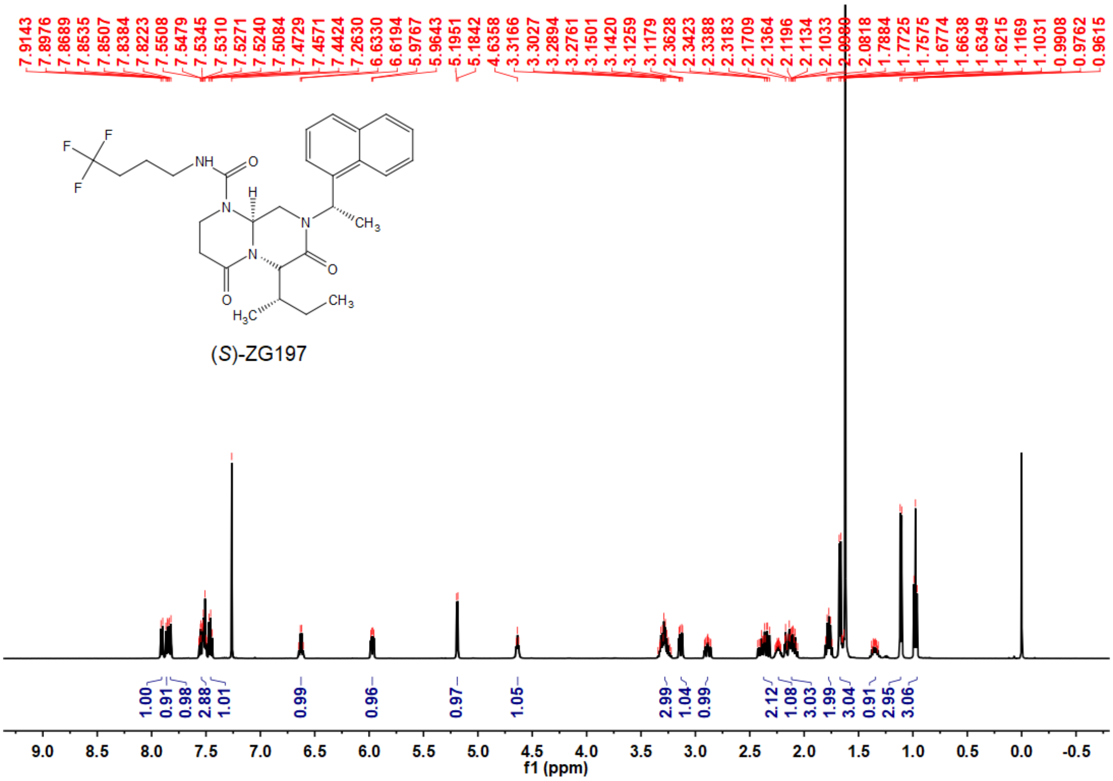


**Supplementary Fig. 11 $^{1}$H NMR-spectrum (500 MHz, $CDCl_3$) of (*S*)-ZG197**

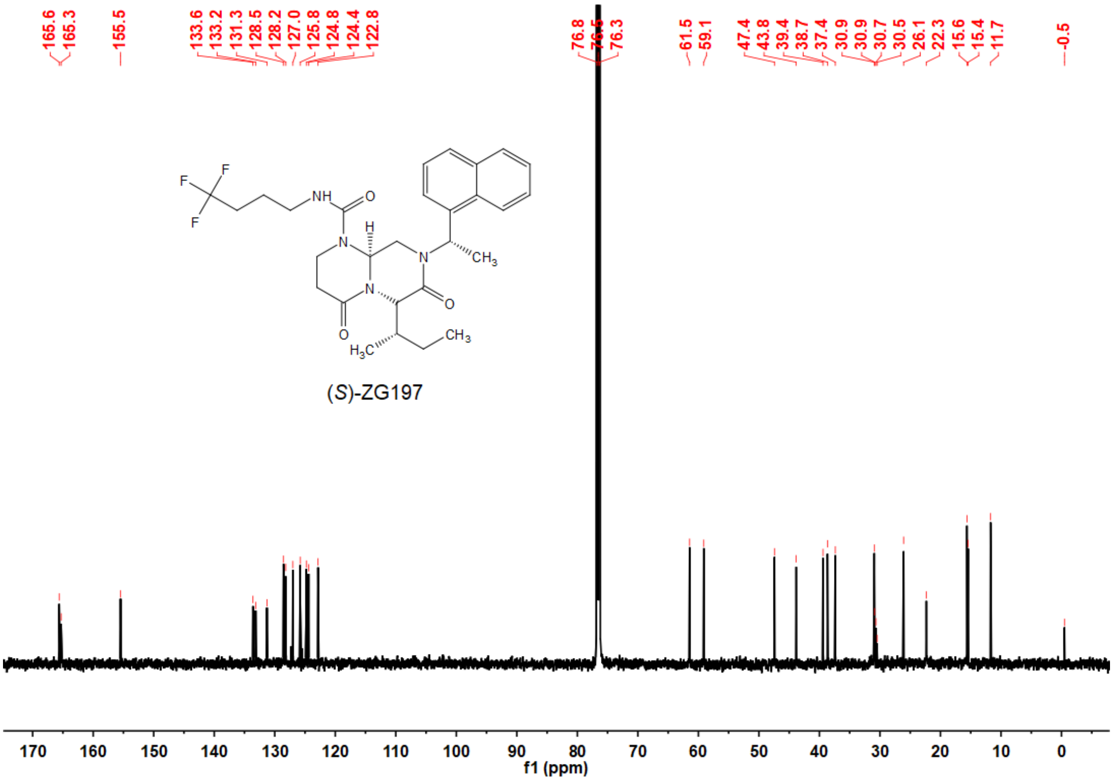


**Supplementary Fig. 12 $^{13}$C NMR-spectrum (125 MHz, $CDCl_3$) of (*S*)-ZG197**

## The purities of ZG180, (*R*)- and (*S*)-ZG197 analyzed by HPLC

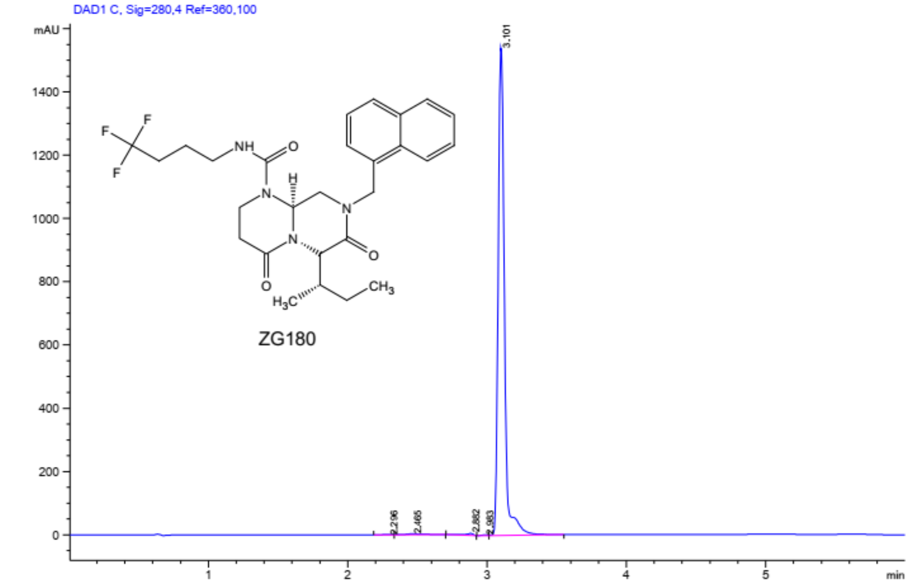


Integration Results for DAD1 C, Sig=280,4 Ref=360,100

| RetTim | Width | Area | Height | Area% |
|---|---|---|---|---|
| 2.30 | 0.09 | 6.75 | 1.25 | 0.14 |
| 2.47 | 0.14 | 30.35 | 3.01 | 0.62 |
| 2.88 | 0.07 | 32.96 | 6.85 | 0.67 |
| 2.98 | 0.06 | 8.43 | 2.30 | 0.17 |
| 3.10 | 0.05 | 4833.59 | 1555.43 | 98.40 |

**Supplementary Fig. 13 The purity of ZG180 analyzed by HPLC**

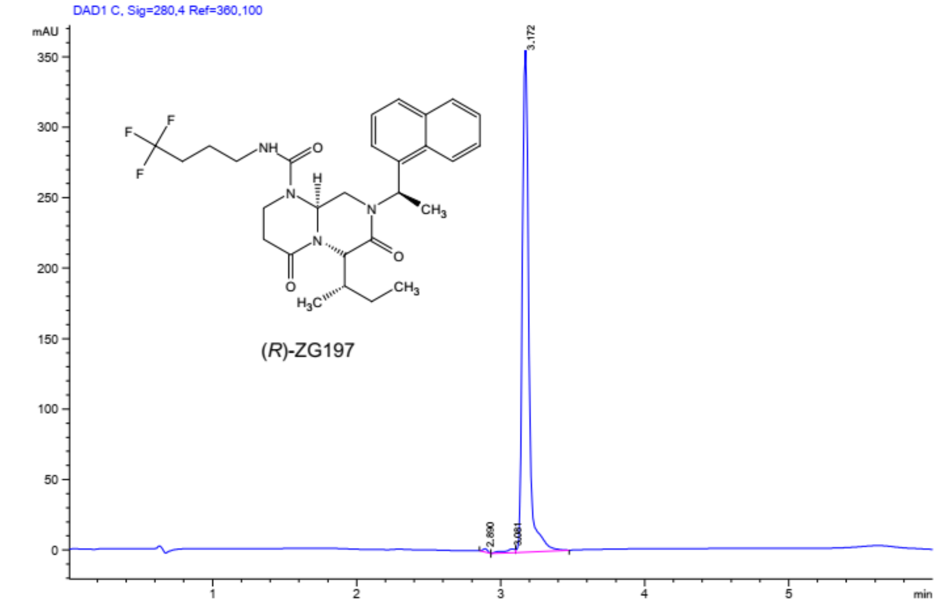


Integration Results for DAD1 C, Sig=280,4 Ref=360,100

| RetTim | Width | Area | Height | Area% |
|---|---|---|---|---|
| 2.89 | 0.04 | 5.65 | 2.42 | 0.49 |
| 3.08 | 0.08 | 14.62 | 2.57 | 1.28 |
| 3.17 | 0.05 | 1122.10 | 356.28 | 98.23 |

**Supplementary Fig. 14 The purity of (*R*)-ZG197 analyzed by HPLC**

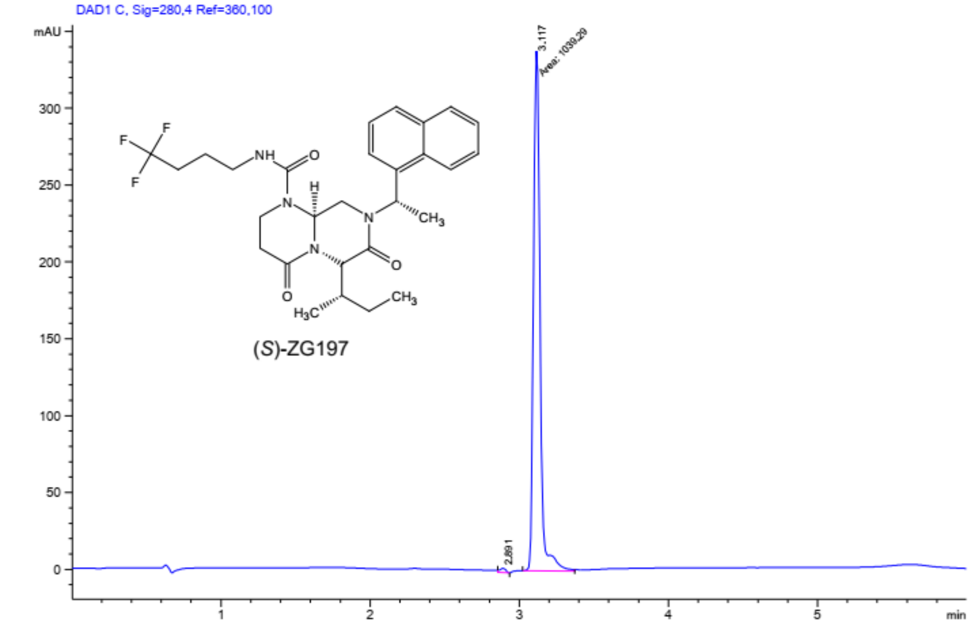


Integration Results for DAD1 C, Sig=280,4 Ref=360,100

| RetTim | Width | Area | Height | Area% |
|---|---|---|---|---|
| 2.89 | 0.04 | 7.85 | 2.82 | 0.75 |
| 3.12 | 0.05 | 1039.29 | 340.02 | 99.25 |

**Supplementary Fig. 15 The purity of (*S*)-ZG197 analyzed by HPLC**

**Chiral purities of (*R*)- and (*S*)-ZG197 determined by four chiral columns**

| | | | |
|---|---|---|---|
| Sample Name: | ZG197-R | Sample Set Name: | AS11 |
| Column Name: | AD-3 4.6*100mm 3um | Processing Method: | AS1 |
| Acq. Method Set: | AD 10% B1 | Vial: | 1:F,7 |
| Co_Solvent: | MeOH[0.2%NH3(7M in MeOH)] | Injection Volume: | 1.00 ul |
| Channel Name: | PDA Ch2 214nm@4.8nm | Run Time: | 6.0 Minutes |
| Proc. Chnl. Descr.: | PDA Ch2 214nm@4.8nm | Flow_rate: | 3.0 mL/min |
| Date Acquired: | 1/12/2022 7:58:32 PM CST | Back_Pressure: | 2000 psi |
| Date Processed: | 1/13/2022 9:40:18 AM CST | Column_Temperature: | 40°C |

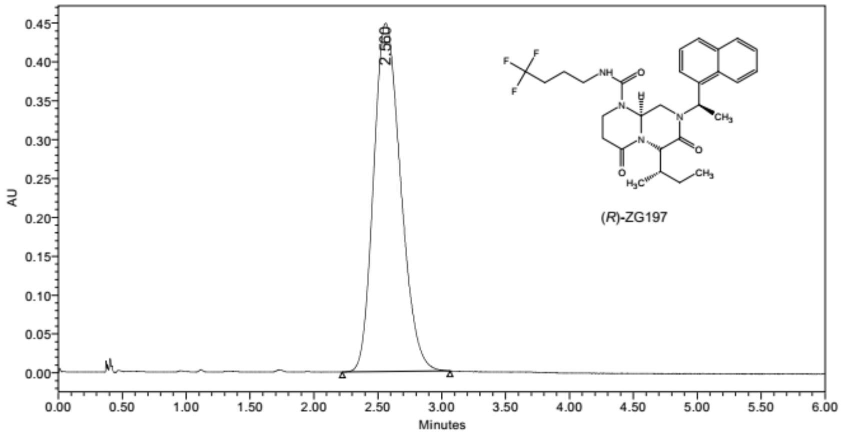


| | RT | Area | % Area | Height |
|---|---|---|---|---|
| 1 | 2.560 | 6608544 | 100.00 | 448127 |

**Supplementary Fig. 16 The chiral purity of (*R*)-ZG197 determined by AD column.**

| | | | |
|---|---|---|---|
| Sample Name: | ZG197-R | Sample Set Name: | AS11 |
| Column Name: | AS-3 4.6*100mm 3um | Processing Method: | AS1 |
| Acq. Method Set: | AS 10% B1 | Vial: | 1:F,7 |
| Co_Solvent: | MeOH[0.2%NH3(7M in MeOH)] | Injection Volume: | 1.00 ul |
| Channel Name: | PDA Ch2 214nm@4.8nm | Run Time: | 6.0 Minutes |
| Proc. Chnl. Descr.: | PDA Ch2 214nm@4.8nm | Flow_rate: | 3.0 mL/min |
| Date Acquired: | 1/12/2022 6:09:35 PM CST | Back_Pressure: | 2000 psi |
| Date Processed: | 1/13/2022 9:39:37 AM CST | Column_Temperature: | 40°C |

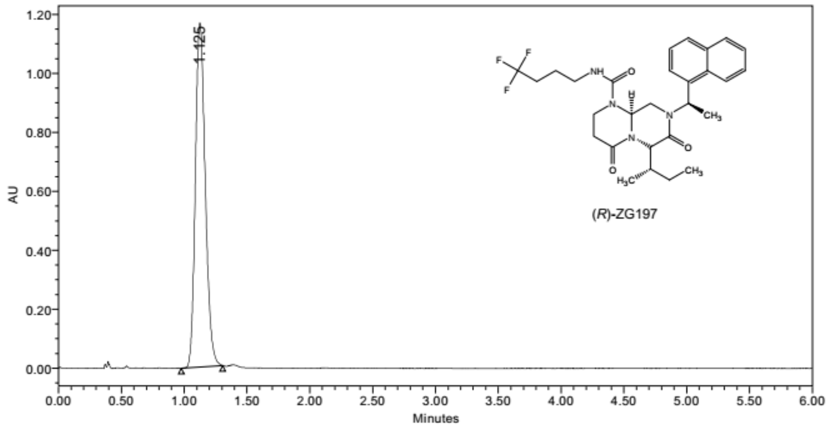


| | RT | Area | % Area | Height |
|---|---|---|---|---|
| 1 | 1.125 | 6366117 | 100.00 | 1166491 |

**Supplementary Fig. 17 The chiral purity of (*R*)-ZG197 determined by AS column.**

| | | | |
|---|---|---|---|
| Sample Name: | ZG197-R | Sample Set Name: | AS11 |
| Column Name: | OD-3 4.6*100mm 3um | Processing Method: | AS1 |
| Acq. Method Set: | OD 15% B1 | Vial: | 1:F,7 |
| Co_Solvent: | MeOH[0.2%NH3(7M in MeOH)] | Injection Volume: | 1.00 ul |
| Channel Name: | PDA Ch2 214nm@4.8nm | Run Time: | 6.0 Minutes |
| Proc. Chnl. Descr.: | PDA Ch2 214nm@4.8nm | Flow_rate: | 3.0 mL/min |
| Date Acquired: | 1/13/2022 10:44:33 AM CST | Back_Pressure: | 2000 psi |
| Date Processed: | 1/13/2022 1:15:41 PM CST | Column_Temperature: | 40°C |

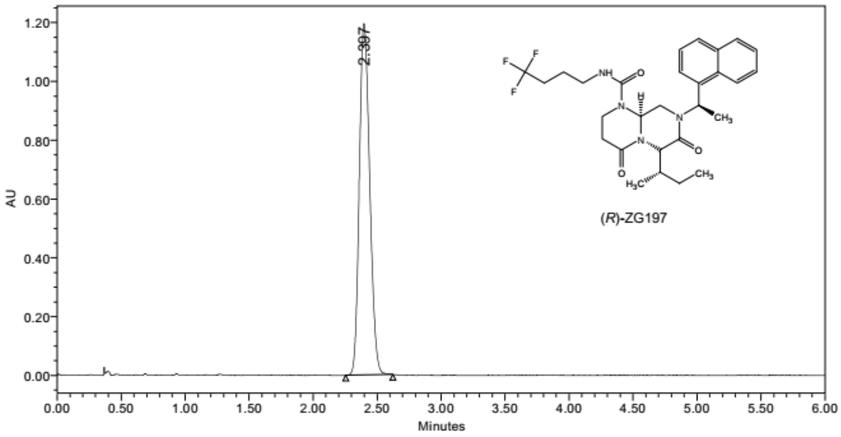


| | RT | Area | % Area | Height |
|---|---|---|---|---|
| 1 | 2.397 | 6789070 | 100.00 | 1195380 |

**Supplementary Fig. 18 The chiral purity of (*R*)-ZG197 determined by OD column.**

| | | | |
|---|---|---|---|
| Sample Name: | ZG197-R | Sample Set Name: | AS11 |
| Column Name: | OJ-3 4.6*100mm 3um | Processing Method: | AS1 |
| Acq. Method Set: | OJ 5% B1 | Vial: | 1:F,7 |
| Co_Solvent: | MeOH[0.2%NH3(7M in MeOH)] | Injection Volume: | 1.00 ul |
| Channel Name: | PDA Ch2 214nm@4.8nm | Run Time: | 6.0 Minutes |
| Proc. Chnl. Descr.: | PDA Ch2 214nm@4.8nm | Flow_rate: | 3.0 mL/min |
| Date Acquired: | 1/13/2022 10:27:31 AM CST | Back_Pressure: | 2000 psi |
| Date Processed: | 1/13/2022 1:15:46 PM CST | Column_Temperature: | 40°C |

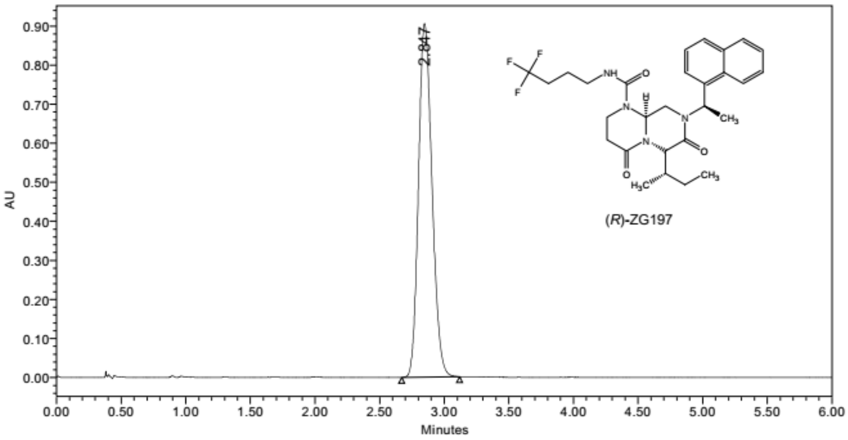


| | RT | Area | % Area | Height |
|---|---|---|---|---|
| 1 | 2.847 | 6582141 | 100.00 | 905844 |

**Supplementary Fig. 19 The chiral purity of (*R*)-ZG197 determined by OJ column.**

| | | | |
|---|---|---|---|
| Sample Name: | ZG197-S | Sample Set Name: | AS11 |
| Column Name: | AD-3 4.6*100mm 3um | Processing Method: | AS1 |
| Acq. Method Set: | AD 10% B1 | Vial: | 1:F,8 |
| Co_Solvent: | MeOH[0.2%NH3(7M in MeOH)] | Injection Volume: | 1.00 ul |
| Channel Name: | PDA Ch2 214nm@4.8nm | Run Time: | 6.0 Minutes |
| Proc. Chnl. Descr.: | PDA Ch2 214nm@4.8nm | Flow_rate: | 3.0 mL/min |
| Date Acquired: | 1/12/2022 8:05:25 PM CST | Back_Pressure: | 2000 psi |
| Date Processed: | 1/13/2022 9:40:48 AM CST | Column_Temperature: | 40°C |

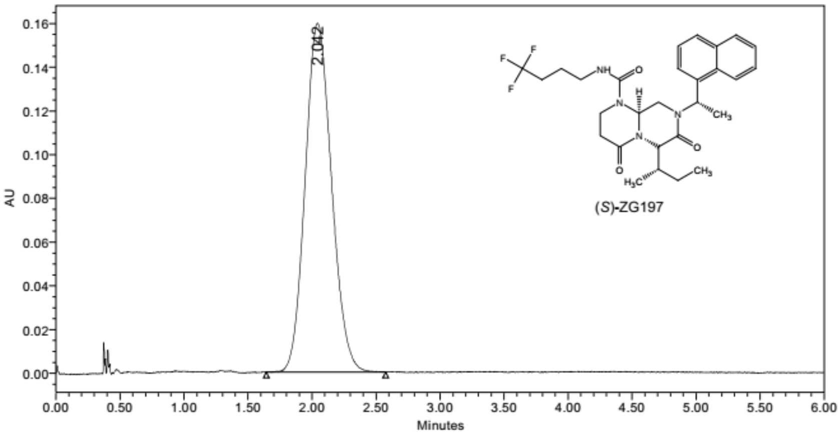


| | RT | Area | % Area | Height |
|---|---|---|---|---|
| 1 | 2.042 | 2288177 | 100.00 | 159578 |

**Supplementary Fig. 20 The chiral purity of (*S*)-ZG197 determined by AD column.**

| | | | |
|---|---|---|---|
| Sample Name: | ZG197-S | Sample Set Name: | AS11 |
| Column Name: | AS-3 4.6*100mm 3um | Processing Method: | AS1 |
| Acq. Method Set: | AS 5% B1 | Vial: | 1:F,8 |
| Co_Solvent: | MeOH[0.2%NH3(7M in MeOH)] | Injection Volume: | 5.00 ul |
| Channel Name: | PDA Ch2 214nm@4.8nm | Run Time: | 6.0 Minutes |
| Proc. Chnl. Descr.: | PDA Ch2 214nm@4.8nm | Flow_rate: | 3.0 mL/min |
| Date Acquired: | 1/13/2022 9:57:54 AM CST | Back_Pressure: | 2000 psi |
| Date Processed: | 1/13/2022 1:16:51 PM CST | Column_Temperature: | 40°C |

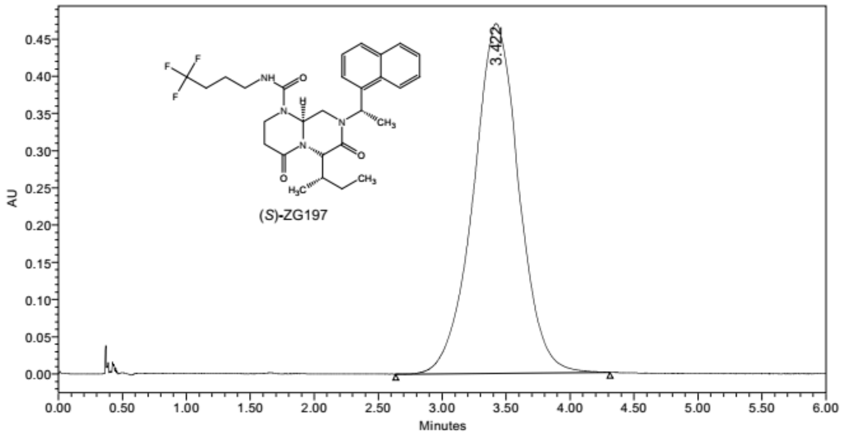


| | RT | Area | % Area | Height |
|---|---|---|---|---|
| 1 | 3.422 | 11647258 | 100.00 | 470103 |

**Supplementary Fig. 21 The chiral purity of (*S*)-ZG197 determined by AS column.**

| | | | |
|---|---|---|---|
| Sample Name: | ZG197-S | Sample Set Name: | AS11 |
| Column Name: | OD-3 4.6*100mm 3um | Processing Method: | AS1 |
| Acq. Method Set: | OD 10% B1 | Vial: | 1:F,8 |
| Co_Solvent: | MeOH[0.2%NH3(7M in MeOH)] | Injection Volume: | 1.00 ul |
| Channel Name: | PDA Ch2 214nm@4.8nm | Run Time: | 6.0 Minutes |
| Proc. Chnl. Descr.: | PDA Ch2 214nm@4.8nm | Flow_rate: | 3.0 mL/min |
| Date Acquired: | 1/12/2022 7:48:25 PM CST | Back_Pressure: | 2000 psi |
| Date Processed: | 1/13/2022 9:40:45 AM CST | Column_Temperature: | 40°C |

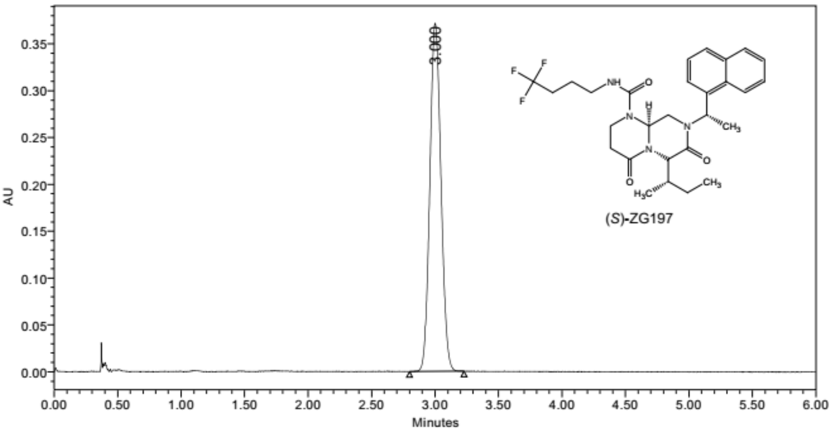


| | RT | Area | % Area | Height |
|---|---|---|---|---|
| 1 | 3.000 | 2269820 | 100.00 | 371739 |

**Supplementary Fig. 22 The chiral purity of (*S*)-ZG197 determined by OD column.**

| | | | |
|---|---|---|---|
| Sample Name: | ZG197-S | Sample Set Name: | AS11 |
| Column Name: | OJ-3 4.6*100mm 3um | Processing Method: | AS1 |
| Acq. Method Set: | OJ 5% B1 | Vial: | 1:F,8 |
| Co_Solvent: | MeOH[0.2%NH3(7M in MeOH)] | Injection Volume: | 1.00 ul |
| Channel Name: | PDA Ch2 214nm@4.8nm | Run Time: | 6.0 Minutes |
| Proc. Chnl. Descr.: | PDA Ch2 214nm@4.8nm | Flow_rate: | 3.0 mL/min |
| Date Acquired: | 1/13/2022 10:34:23 AM CST | Back_Pressure: | 2000 psi |
| Date Processed: | 1/13/2022 1:15:43 PM CST | Column_Temperature: | 40°C |

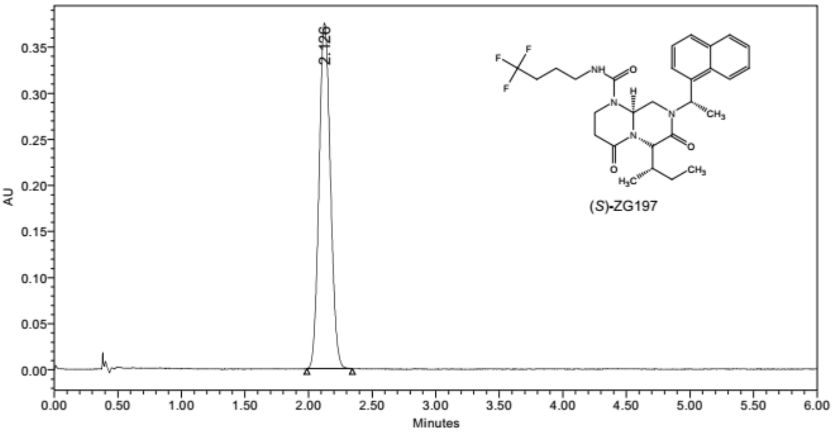


| | RT | Area | % Area | Height |
|---|---|---|---|---|
| 1 | 2.126 | 2316926 | 100.00 | 374803 |

**Supplementary Fig. 23 The chiral purity of (*S*)-ZG197 determined by OJ column.**

**Supplementary References**


1. Novick R. Properties of a cryptic high-frequency transducing phage in Staphylococcus aureus. *Virology* **33**, 155-166 (1967).
2. Frees D, Qazi SN, Hill PJ, et al. Alternative roles of ClpX and ClpP in Staphylococcus aureus stress tolerance and virulence. *Mol Microbiol* **48**, 1565-1578 (2003).
3. Duthie ES, Lorenz LL. Staphylococcal coagulase; mode of action and antigenicity. *J Gen Microbiol* **6**, 95-107 (1952).
4. Ni T, Ye F, Liu X, et al. Characterization of Gain-of-Function Mutant Provides New Insights into ClpP Structure. *ACS Chem Biol* **11**, 1964-1972 (2016).
5. Kreiswirth BN, Löfdahl S, Betley MJ, et al. The toxic shock syndrome exotoxin structural gene is not detectably transmitted by a prophage. *Nature* **305**, 709-712 (1983).
6. Bubeck Wardenburg J, Schneewind O. Vaccine protection against Staphylococcus aureus pneumonia. *J Exp Med* **205**, 287-294 (2008).
7. Kuroda M, Ohta T, Uchiyama I, et al. Whole genome sequencing of meticillin-resistant Staphylococcus aureus. *Lancet* **357**, 1225-1240 (2001).
8. de Lencastre H, Tomasz A. Reassessment of the number of auxiliary genes essential for expression of high-level methicillin resistance in Staphylococcus aureus. *Antimicrob Agents Chemother* **38**, 2590-2598 (1994).
9. Lelièvre H, Lina G, Jones ME, et al. Emergence and spread in French hospitals of methicillin-resistant Staphylococcus aureus with increasing susceptibility to gentamicin and other antibiotics. *J Clin Microbiol* **37**, 3452-3457 (1999).
10. Wilson P, Andrews JA, Charlesworth R, et al. Linezolid resistance in clinical isolates of Staphylococcus aureus. *J Antimicrob Chemother* **51**, 186-188 (2003).
11. Wang P, Zhang T, Wang X, et al. Aberrant human ClpP activation disturbs mitochondrial proteome homeostasis to suppress pancreatic ductal adenocarcinoma. *Cell Chem Biol*, **29**, 1396-1408.e8 (2022).
