# Supplementary material for: Anti-infective therapy using species-specific activators of Staphylococcus aureus ClpP
Source: Nat Commun. 2022 Nov 14;13:6909. doi: 10.1038/s41467-022-34753-0 (PMC9663597; doi:10.1038/s41467-022-34753-0)
Supplement: Supplementary file 3 — Reporting Summary [file 41467_2022_34753_MOESM3_ESM.pdf]

nature portfolio

Corresponding author(s): Cai-Guang Yang

Last updated by author(s): Nov 3, 2022

Reporting Summary

Nature Portfolio wishes to improve the reproducibility of the work that we publish. This form provides structure for consistency and transparency in reporting. For further information on Nature Portfolio policies, see our Editorial Policies and the Editorial Policy Checklist.

Statistics

For all statistical analyses, confirm that the following items are present in the figure legend, table legend, main text, or Methods section.

n/a	Confirmed	
☐	☒	The exact sample size (*n*) for each experimental group/condition, given as a discrete number and unit of measurement
☐	☒	A statement on whether measurements were taken from distinct samples or whether the same sample was measured repeatedly
☐	☒	The statistical test(s) used AND whether they are one- or two-sided<br>*Only common tests should be described solely by name; describe more complex techniques in the Methods section.*
☒	☐	A description of all covariates tested
☒	☐	A description of any assumptions or corrections, such as tests of normality and adjustment for multiple comparisons
☐	☒	A full description of the statistical parameters including central tendency (e.g. means) or other basic estimates (e.g. regression coefficient) AND variation (e.g. standard deviation) or associated estimates of uncertainty (e.g. confidence intervals)
☐	☒	For null hypothesis testing, the test statistic (e.g. *F*, *t*, *r*) with confidence intervals, effect sizes, degrees of freedom and *P* value noted<br>*Give P values as exact values whenever suitable.*
☒	☐	For Bayesian analysis, information on the choice of priors and Markov chain Monte Carlo settings
☒	☐	For hierarchical and complex designs, identification of the appropriate level for tests and full reporting of outcomes
☒	☐	Estimates of effect sizes (e.g. Cohen's *d*, Pearson's *r*), indicating how they were calculated

Our web collection on statistics for biologists contains articles on many of the points above.

Software and code

Policy information about availability of computer code

Data collection	Biolayer interferometry (BLI) assay data were collected by using Octet Acquisition 11.0.<br>Differential scanning fluorimetry (DSF) data collection were collected using CFX manager 3.1.<br>Isothermal titration calorimetry (ITC) data collection were performed using MicroCal iTC200.<br>Cytotoxicity assay data were collected by Tecon SPARKCONTROL v2.2<br>Diffraction data of ZG180/SaClpP and (R)-ZG197/SaClpP were collected via Bluice 5.0.<br>Diffraction data of ZG180/HsClpP or (S)-ZG197/SaClpP were collected via Finback 1.0.
Data analysis	All statistical analysis were performed in GraphPad Prism v8.2.1.<br>Quantitative analysis were performed using ImageJ 1.52v.<br>ITC data were performed using OriginPro 8.5 ITC.<br>Molecular alignments were performed in PyMOL v2.4+.<br>The sequence alignment was performed using ClustalW and ENDscript/ESPript.<br>The models of SaClpP (PDB code 3STA) and HsClpP (PDB code 1TG6) were built in COOT 0.8.1.<br>Diffraction data of ZG180/SaClpP and (R)-ZG197/SaClpP were processed via HKL2000 program suite and refined with the program REFMAC5.<br>Diffraction data of ZG180/HsClpP or (S)-ZG197/SaClpP were automatically processed by Aquarium and refined with the program REFMAC5.<br>The Biolayer interferometry (BLI) assay data were analysed using Octet Analysis 11.0.<br>Differential scanning fluorimetry (DSF) data analysis were performed with Bio-Rad CFX Manager 3.1.<br>Graphs of scanning electron microscopy data were adjusted using Adobe Photoshop CS5.

For manuscripts utilizing custom algorithms or software that are central to the research but not yet described in published literature, software must be made available to editors and reviewers. We strongly encourage code deposition in a community repository (e.g. GitHub). See the Nature Portfolio guidelines for submitting code & software for further information.

Data

Policy information about availability of data

All manuscripts must include a data availability statement. This statement should provide the following information, where applicable:

- Accession codes, unique identifiers, or web links for publicly available datasets
- A description of any restrictions on data availability
- For clinical datasets or third party data, please ensure that the statement adheres to our policy

The atomic coordinates and structure factors data generated in this study have been deposited in the Protein Data Bank (PDB, www.pdb.org) under accession code 7WH5 [http://doi.org/10.2210/pdb7WH5/pdb] for ZG180/HsClpP; 7WID [http://doi.org/10.2210/pdb7WID/pdb] for ZG180/SaClpP; 7XBZ [http://doi.org/10.2210/pdb7XBZ/pdb] for (R)-ZG197/SaClpP; and 7WGS [http://doi.org/10.2210/pdb7WGS/pdb] for (S)-ZG197/SaClpP structure, respectively. Other X-ray structural data used in this study are available in the PDB database under accession code 6TTY [http://doi.org/10.2210/pdb6TTY/pdb], 6TTZ [http://doi.org/10.2210/pdb6TTZ/pdb], 3STA [http://doi.org/10.2210/pdb3STA/pdb] and 1TG6 [http://doi.org/10.2210/pdb1TG6/pdb]. Amino acid sequence can be found at National Center for Biotechnology Information (NCBI, https://www.ncbi.nlm.nih.gov/) with the accession number of NP_006003 [https://www.ncbi.nlm.nih.gov/protein/NP_006003.1] for HsClpP; CAA06443 [https://www.ncbi.nlm.nih.gov/protein/CAA06443.1] for MoClpP; NP_001018520 [https://www.ncbi.nlm.nih.gov/protein/NP_001018520.1] for DaClpP; KFL07692 [https://www.ncbi.nlm.nih.gov/protein/KFL07692.1] for SaClpP; and CAD6014684 [https://www.ncbi.nlm.nih.gov/protein/CAD6014684.1] for EcClpP. All relevant data generated in this study are provided in the Supplementary Information and Source Data file. Source Data are provided with this paper.

Field-specific reporting

Please select the one below that is the best fit for your research. If you are not sure, read the appropriate sections before making your selection.

[x] Life sciences [ ] Behavioural & social sciences [ ] Ecological, evolutionary & environmental sciences

For a reference copy of the document with all sections, see nature.com/documents/nr-reporting-summary-flat.pdf

Life sciences study design

All studies must disclose on these points even when the disclosure is negative.

Sample size	No statistical methods were used to determine sample sizes. Sample sizes were determined on the basis of previous experimental experience (Nat Commun. 2018, 9, 5102; Cell. 2020, 181, 1518-1532.e14; PLoS Pathog. 2012, 8, e1002944). Sample sizes were sufficient to perform statistical analyses.
Data exclusions	No samples were excluded.
Replication	The number of independent experiments/mice/samples are mentioned in the figure legends. All attempts at replication are successful. All blots and gels were performed in triplicate and a single experimental image is shown. Murine skin infection experiments, SEM and H&E staining were performed once. At least 5 representative images of SEM were recorded from each sample. Three representative images of H&E staining were taken in each sample and one is shown. All images recorded on SEM or H&E staining indicated a similar trend.
Randomization	All zebrafish or mice were first infected with bacteria and randomly allocated into different groups. Other samples, such as biochemical, cell or microorganism samples were maintained or cultured in the same environment and randomly allocated into different groups.
Blinding	Quantification on bacterial counts in the murine skin samples were performed blindly. The investigators did not know the group information of each samples. Other experiments were not performed blindly because these results can be directly and easily obtained from instrument measurements or visual observations.

Reporting for specific materials, systems and methods

We require information from authors about some types of materials, experimental systems and methods used in many studies. Here, indicate whether each material, system or method listed is relevant to your study. If you are not sure if a list item applies to your research, read the appropriate section before selecting a response.

Materials & experimental systems

n/a	Involved in the study	
[ ]	[x]	Antibodies
[ ]	[x]	Eukaryotic cell lines
[x]	[ ]	Palaeontology and archaeology
[ ]	[x]	Animals and other organisms
[x]	[ ]	Human research participants
[x]	[ ]	Clinical data
[x]	[ ]	Dual use research of concern

Methods

n/a	Involved in the study	
[x]	[ ]	ChIP-seq
[x]	[ ]	Flow cytometry
[x]	[ ]	MRI-based neuroimaging

Antibodies

Antibodies used	Anti-SaClpP (Cat# C11185, 1:5,000), anti-SaFtsZ (Cat# C11186, 1:5,000) and anti-SaGAPDH (Cat# C1399, 1:5,000) were generated by Shanghai Immune Biotech Ltd using the purified protein as the antigen.<br>Anti-HsClpP (Abcam, Cat# ab124822, Clo# EPR7133, Lot# GR3210822-8, 1:2,000)<br>Anti-β-Actin (Proteintech, Cat# 66009-1-Ig, Clo# 2D4H5, Lot# 10004156, 1:5,000)<br>HRP-conjugated goat anti-rabbit IgG (Cwbio, Cat# CW0103, 1:10,000)<br>HRP-conjugated goat anti-mouse IgG (Cwbio, Cat# CW0102, 1:10,000)
Validation	Anti-SaClpP, anti-SaFtsZ and anti-SaGAPDH were validated by manufacturer using Elisa experiments. The validation reports are provided by the manufacturer and are available on request. All of the three antibodies have been utilized for Western Blot in the previous publications from our lab (ACS Chem. Biol. 2016, 11, 1964-1972).<br>Anti-HsClpP, anti-β-Actin, HRP-conjugated goat anti-rabbit IgG and HRP-conjugated goat anti-mouse IgG were purchased from the supplier, and used without additional validation. The validation of all the antibodies can be found on the manufacturers' websites:<br>Anti-HsClpP (https://www.abcam.cn/clpp-antibody-epr7133-ab124822.html)<br>Anti-β-Actin (https://www.ptgcn.com/products/Pan-Actin-Antibody-66009-1-Ig.htm)<br>HRP-conjugated goat anti-rabbit IgG (Cwbio, https://www.cwbio.com/goods/index/id/10119)<br>HRP-conjugated goat anti-mouse IgG (Cwbio, https://www.cwbio.com/goods/index/id/10118)

Eukaryotic cell lines

Policy information about cell lines

Cell line source(s)	HEK 293T/17 and HK-2 cells were purchased from American Type Culture Collection.
Authentication	HEK 293T/17 and HK-2 cells were not authenticated.
Mycoplasma contamination	HEK 293T/17 and HK-2 cell lines were tested negative for mycoplasma contamination.
Commonly misidentified lines (See ICLAC register)	No commonly misidentified cell lines were used in this study.

Animals and other organisms

Policy information about studies involving animals; ARRIVE guidelines recommended for reporting animal research

Laboratory animals	Wild-type zebrafish (7 to 11 months old, 300 ± 50 mg, regardless of gender) were purchased from the Xiaoguan aquarium (Shanghai, China) and maintained in a 10-L tank at ambient temperature with regular feeding. Female BALB/c mice (6 to 8 weeks old, 18-20 g) were purchased from Zhejiang Vital River Laboratory Animal Technology Co., Ltd. S. aureus NCTC 8325-4 and 8325-4/ΔclpP were kindly provided by Hanne Ingmer lab (Royal Veterinary and Agricultural University, Denmark). 8325-4/ΔclpP::clpPI91W, 8325-4/ΔclpP::clpP were constructed in our lab. Clinical MRSA isolates (XJ009, XJ036, XJ049, XJ051, XJ052) were from Wenjuan Wu lab (Shanghai East hospital, China). S. aureus Newman, S. aureus RN4220 and six clinical MRSA strains (USA300 LAC, NRS1, NRS70, NRS100, NRS108, NRS271) were from Lefu Lan lab (Shanghai Institute of Materia Medica, China).
Wild animals	The study did not involve wild animals.
Field-collected samples	No field-collected samples were used in the study.
Ethics oversight	The murine skin infection experiments were performed in accordance with the Institutional Animal Care and Use Committee (IACUC) of Shanghai Public Health Clinical Center.

Note that full information on the approval of the study protocol must also be provided in the manuscript.
